# Supplementary material for: Lysoptosis is an evolutionarily conserved cell death pathway moderated by intracellular serpins
Source: Commun Biol. 2022 Jan 12;5:47. doi: 10.1038/s42003-021-02953-x (PMC8755814; doi:10.1038/s42003-021-02953-x)
Supplement: Supplementary file 2 — Supplementary Information [file 42003_2021_2953_MOESM2_ESM.pdf]

LYSOPTOSIS IS AN EVOLUTIONARY CONSERVED CELL DEATH PATHWAY MODERATED BY INTRACELLULAR SERPINS

SUPPLEMENTARY INFORMATION:

Supplementary Tables and Figures

Cell line	% WT alleles	% gene indels
SW756$^{B3\text{-}WT;CTSL\text{-}KO}$	11.6	88.4
SW756$^{B3\text{-}KO;CTSL\text{-}KO}$	41.7	59.3

Table S1. NGS sequencing results for the cathepsin L knockout lines.

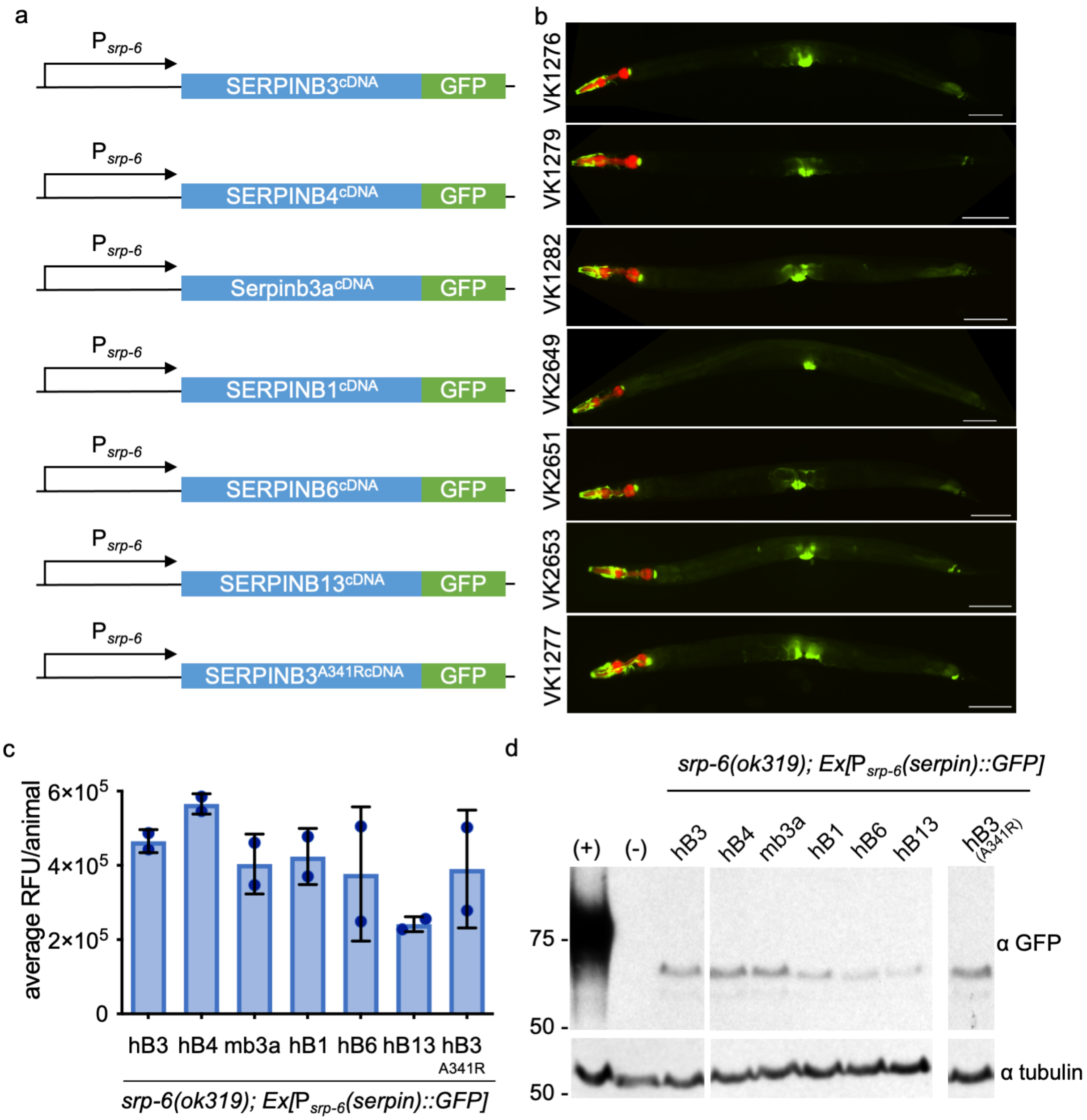
a
P srp-6
SERPINB3cDNA
GFP
SERPINB4cDNA
Serpinb3acDNA
SERPINB1cDNA
SERPINB6cDNA
SERPINB13cDNA
SERPINB3A341RcDNA
b
VK1276
VK1279
VK1282
VK2649
VK2651
VK2653
VK1277
c
average RFU/animal
6×10^5
4×10^5
2×10^5
0
hB3 hB4 mb3a hB1 hB6 hB13 hB3 A341R
srp-6(ok319); Ex[Psrp-6(serpin)::GFP]
d
srp-6(ok319); Ex[Psrp-6(serpin)::GFP]
(+) (-) hB3 hB4 mb3a hB1 hB6 hB13 hB3 (A341R)
75 -
50 -
α GFP
α tubulin
50 -

Supplementary Figure 1. Some mammalian serpins can replace the function of SRP-6 in *C. elegans*. (a) Schematic representation of the constructs used to generate the corresponding transgenic *C. elegans*. (b) Fluorescence microscopy of representative transgenic *C. elegans,* VK1276 (*srp-6(ok319);vkEx1276[*$P_{srp\text{-}6}$*SERPINB3*^{cDNA}*::GFP;* $P_{myo\text{-}2}$*mCherry])* (hB3), VK1279 (*srp-6(ok319);vkEx1279[*$P_{srp\text{-}6}$*SERPINB4*^{cDNA}*::GFP;* $P_{myo\text{-}2}$*mCherry])* (hB4), VK1282 (*srp-6(ok319);vkEx1282[*$P_{srp\text{-}6}$*Serpinb3a*^{cDNA}*::GFP;* $P_{myo\text{-}2}$*mCherry])* (mB3a), VK2649 (*srp-6(ok319);vkEx2649[*$P_{srp\text{-}6}$*SERPINB1*^{cDNA}*::GFP;* $P_{myo\text{-}2}$*mCherry])* (hB1), VK2651 (*srp-6(ok319);vkEx2651[*$P_{srp\text{-}6}$*SERPINB6*^{cDNA}*::GFP;* $P_{myo\text{-}2}$*mCherry])* (hB6), VK2653 (*srp-6(ok319);vkEx2653[*$P_{srp\text{-}6}$*SERPINB13*^{cDNA}*::GFP;* $P_{myo\text{-}2}$*mCherry])* (hB13) and VK1277 (*srp-6(ok319);vkEx1276[*$P_{srp\text{-}6}$*SERPINB3*$^{(A341R)cDNA}$*::GFP;* $P_{myo\text{-}2}$*mCherry])* (hB3$^{(A341R)}$) acquired using 488nm and 568nm excitation laser lines to excite GFP (green) and mCherry (red). (c) Approximately 15 animals of each of the transgenic lines were placed in individual wells of a 384-well plate over n = 2 wells and fluorescence was measured using the Cellinsight CX7 imaging plate reader (Thermo Fisher Scientific) in both the GFP (Ex 485 nm / Em 503-531 nm) and RFP (Ex 560 nm / Em 590-624 nm) channels. GFP and mCherry positive objects were identified by thresholding and the relative GFP fluorescence (RFU) was divided by the number of mCherry positive objects to generate the RFU / animal. The average RFU per animal was calculated over the multiple wells. The error bars represent the standard deviation from the mean. (d) Western blot analysis of the serpin transgene expression levels in the *C. elegans* transgenic lines. One hundred transgenic *C. elegans* were lysed in SDS-PAGE sample buffer with boiling for 5 mins and the proteins separated by SDS-PAGE prior to being transferred onto

nitrocellulose. Transgene expression was then detected using anti-GFP mAb. Both N2 (-) and VK694 (P_{nhx-2}*sGFP::ATZ;* +) served as negative and positive western blotting controls. Loading of protein was determined by anti-tubulin mAb loading control.

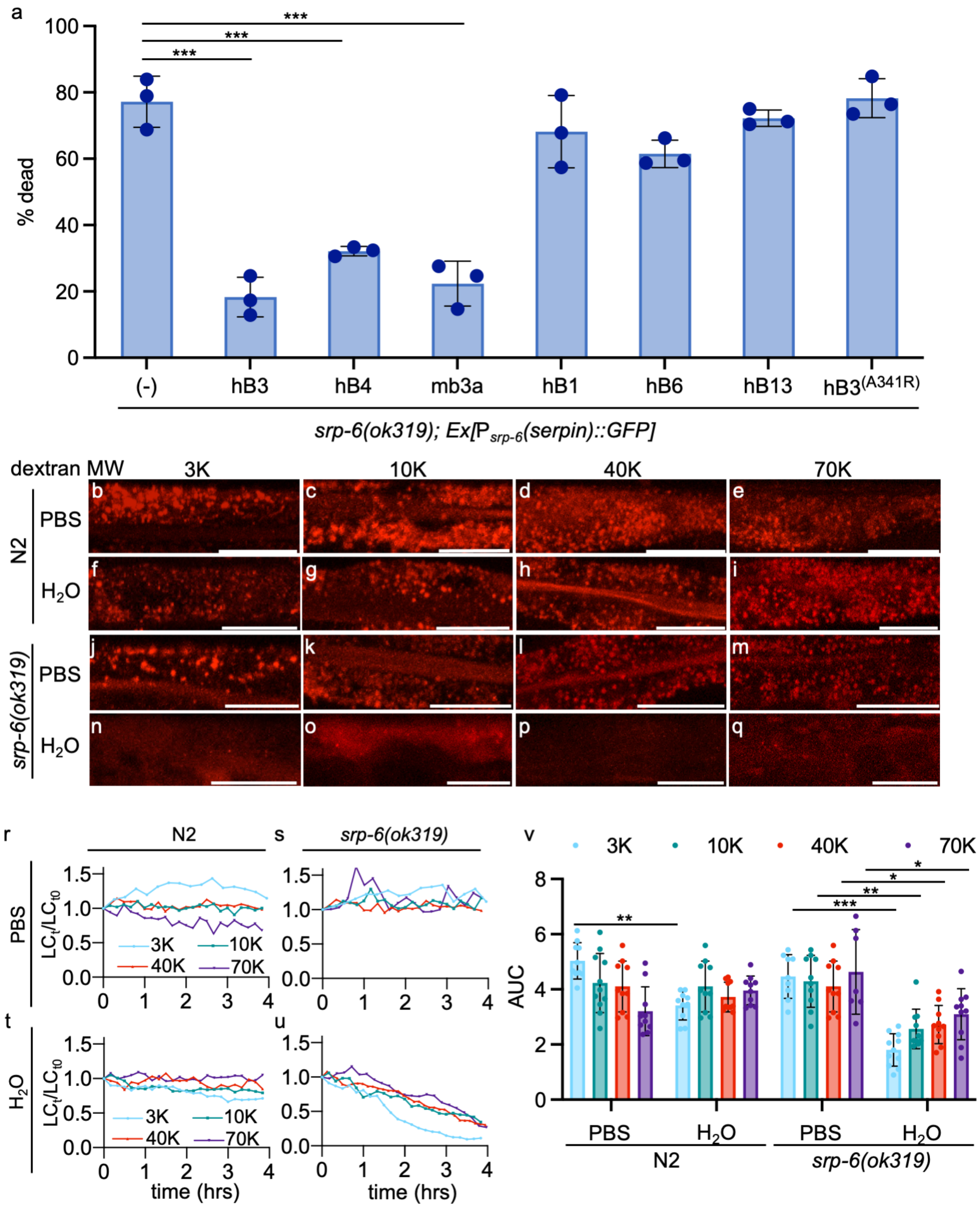
a
% dead
***
***
***
(-)
hB3
hB4
mb3a
hB1
hB6
hB13
hB3(A341R)
srp-6(ok319); Ex[Psrp-6(serpin)::GFP]
dextran MW
3K
10K
40K
70K
N2
PBS
H2O
srp-6(ok319)
r
N2
s
srp-6(ok319)
LCt/LCt0
3K
10K
40K
70K
t
u
time (hrs)
v
AUC
**
***
**
*
*
PBS
H2O
PBS
H2O
N2
srp-6(ok319)

Supplementary Figure 2. Mammalian clade B serpins inhibit lysoptosis in *C. elegans*. (a) Survival of *srp-6(ok319);$P_{myo\text{-}2}$mCherry* (-) and *srp-6(ok319);$P_{myo\text{-}2}$mCherry* transgenic lines expressing human *SERPINB3* (hB3, strain VK1276), human *SERPINB4* (hb4, strain VK1279), mouse *Serpinb3a* (mB3a, strain VK1282), human *SERPINB1* (hB1, strain VK2649), human *SERPINB6* (hB6, strain VK2651), human *SERPINB13* (hB13, strain, VK2653) and human *SERPINB3* with an inactivating A341R mutation (hB3$^{(A341R)}$), strain VK1277) after exposure to hypotonic stress for 1 hour. Strains were selected for comparable levels of protein expression in mosaic animals (Fig. S1). Animals (n ≥ 20) for each group were assayed on 3 separate days. The mean percent dead ± the standard deviation (SD) from all three assays were plotted (****P* < 0.001; two-tailed *t*-test). (b-v) Hypotonic stress induced lysosomal membrane permeabilization (LMP) in *srp-6(ok319) C. elegans*. (b-q). Representative maximum intensity projection confocal images of both wild-type (N2; b-i) and *srp-6(ok319) C. elegans* (j-q) labeled with Texas red conjugated dextrans of different molecular masses (kDa). Animals were exposed to PBS (b-e; j-m) or H_2O (f-i; n-q) and fluorescence (red; Ex 594nm/Em 620nm) was acquired by live animal confocal microscopy in XYZ planes approximately every 5 min over 4 hours in single animals (n ≥ 7). The 4-hour time point is shown. Scale bars = 25 µm. (r-u) Quantification of the different molecular mass dextrans (3 kDa (3K) (cyan), 10K (turquoise), 40K (red) and 70K (purple) in multiple animals (n ≥ 10) of both wild-type N2 (r, t) and *srp-6(ok319)* (s, u) *C. elegans* exposed to PBS (r, s) or H_2O (t, u). The lysosomal count at each time point (LC_t) was normalized to the lysosome count at time point 0 (LC_{t0}) to generate the relative lysosomal abundance (LC_t/LC_{t0}). The average LC_t/LC_{t0} ± SD for each time point was plotted. Error

bars were not included to allow for visualization of the curves. A representative of 3 experiments is shown. (v) The area under the curve (AUC) was calculated for each dextran in each individual animal and the mean AUC ± SD was plotted. The AUCs for the same molecular mass dextrans in the same *C. elegans* strains treated with PBS or H_2O were compared by 2-way ANOVA with Tukey multiple comparisons (***$P < 0.001$, **$P < 0.01$ and *$P < 0.05$). A representative experiment is shown.

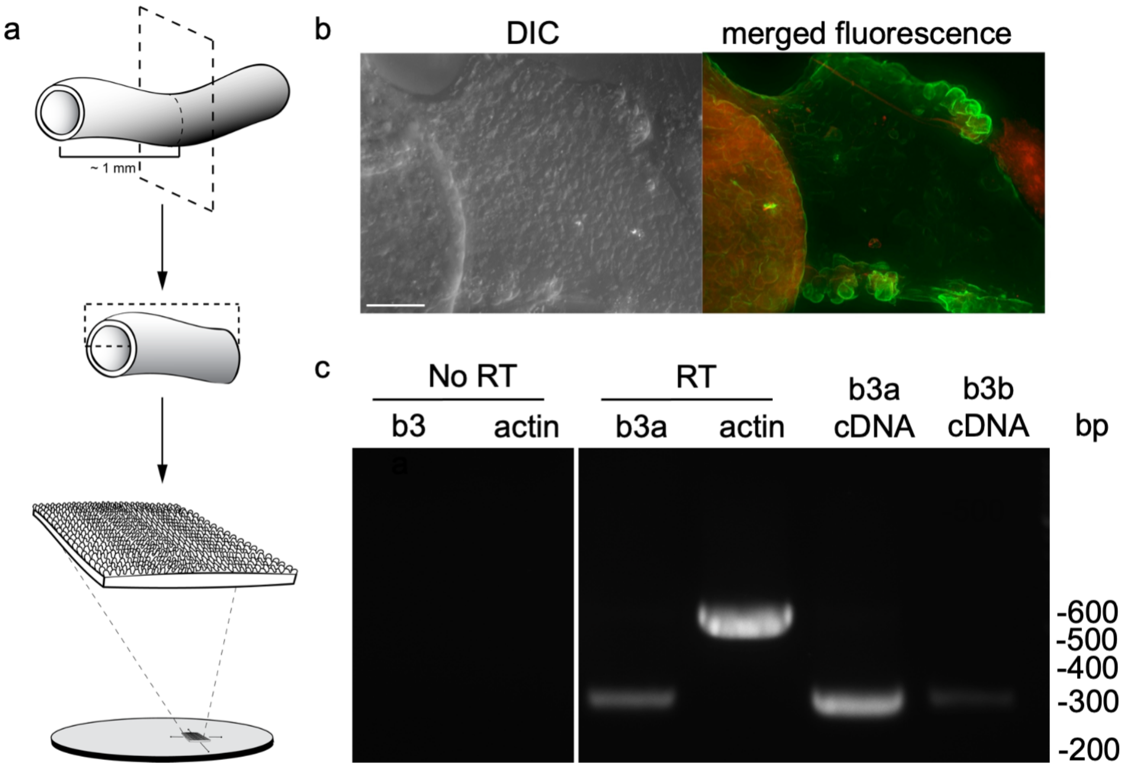
a
~ 1 mm
b
DIC
merged fluorescence
c
No RT
b3
actin
RT
b3a
actin
b3a
cDNA
b3b
cDNA
bp
-600
-500
-400
-300
-200

Supplementary Figure 3. Establishment of FIEC cultures. (a) Schematic representation of the preparation of FIEC cultures for ex vivo imaging experiments. (b) Immunofluorescence of FIECs derived from *mSerpinb3a*$^{+/+}$ mice with a hamster monoclonal antibody that recognizes *Serpinb3a* and *Serpinb3b* (red) and a rabbit polyclonal antibody that recognizes the epithelial cell marker cytokeratin 8 (green). Primary antibodies were detected with Alexa Fluor 594 conjugated goat anti hamster and Alexa Fluor 488 conjugated goat anti-rabbit, respectively. (c) RT-PCR from FIECs derived from *mSerpinb3a*$^{+/+}$ mice with primers specific for mSerpinb3a (expected product 302 bp) and actin (expected product 537 bp). No reverse transcriptase (No RT) reactions served as negative controls for the amplification of genomic DNA. Primer specificity was determined using plasmids containing the cDNA for *mSerpinb3a* (+ve) and *mSerpinb3b* (-ve).

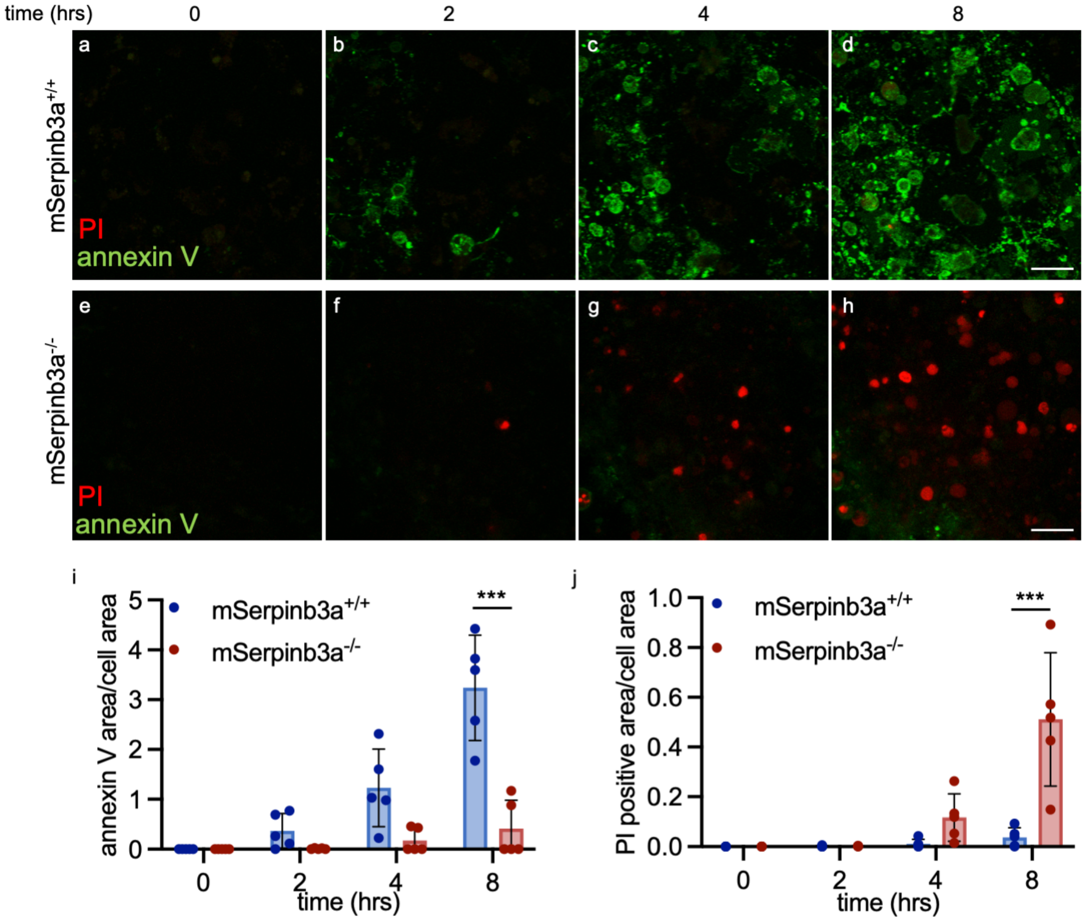
time (hrs)
0
2
4
8
a
b
c
d
mSerpinb3a+/+
PI
annexin V
e
f
g
h
mSerpinb3a-/-
PI
annexin V
i
annexin V area/cell area
mSerpinb3a+/+
mSerpinb3a-/-
***
time (hrs)
j
PI positive area/cell area
mSerpinb3a+/+
mSerpinb3a-/-
***
time (hrs)

Supplementary Figure 4. mSerpinb3a$^{-/-}$ FIECs lose plasma membrane integrity early with staurosporine treatment. (a-h) Representative live cell time-lapse confocal fluorescence maximum intensity projections of mSerpinb3a$^{+/+}$ (a-d) or mSerpinb3a$^{-/-}$ (e-h) FIECs treated with 10 μM staurosporine and stained with annexin V-FITC (green) and propidium iodide (red) at the indicated time points (hrs). Scale bar = 25 μm. (i, j) Quantification of the annexin V area/cell area (i) and PI positive area/cell area (j) from multiple fields of view (n = 5) during the live-cell time lapse imaging at the indicated time points.

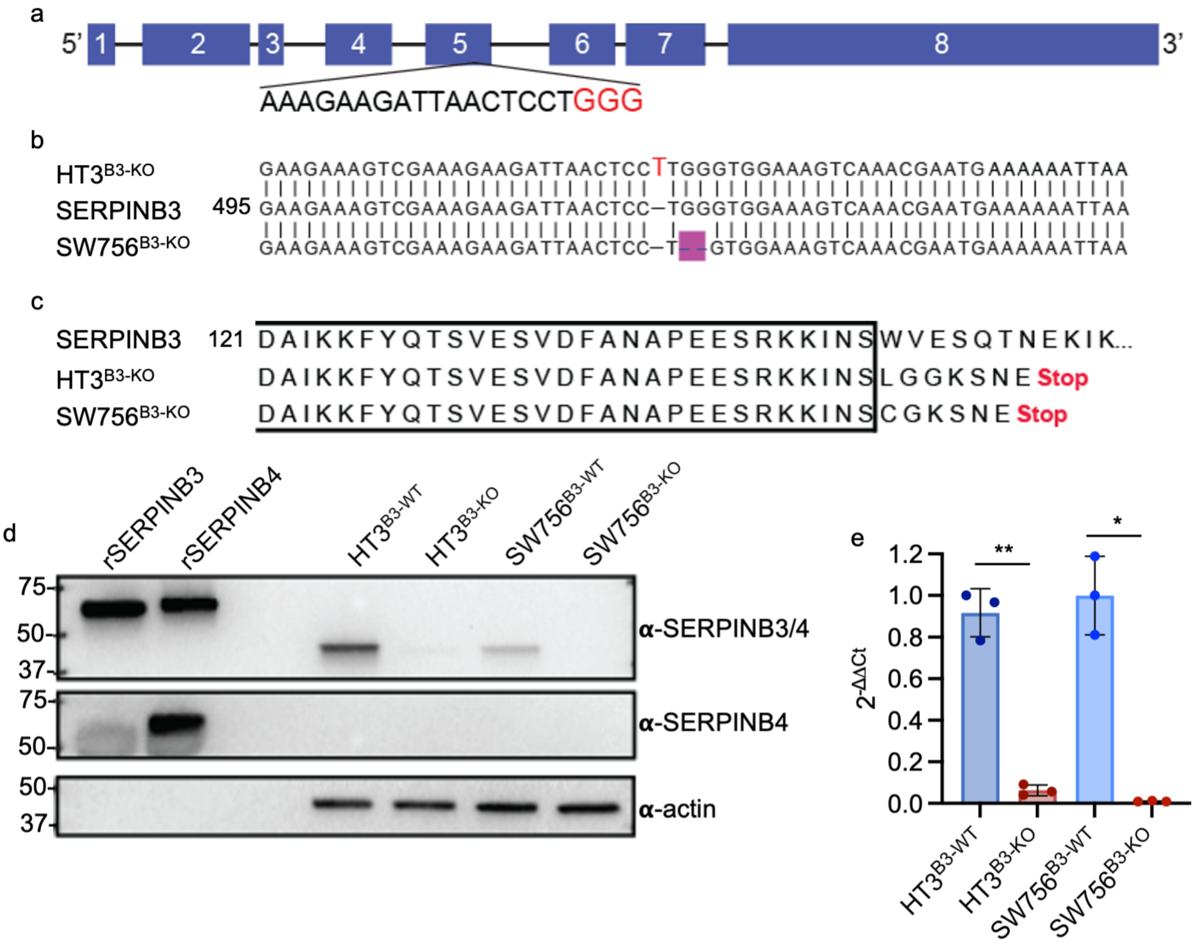
a
5'
1
2
3
4
5
6
7
8
3'
AAAGAAGATTAACTCCTGGG
b
HT3B3-KO
SERPINB3
495
SW756B3-KO
GAAGAAAGTCGAAAGAAGATTAACTCCTTGGGTGGAAAGTCAAACGAATGAAAAAATTAA
GAAGAAAGTCGAAAGAAGATTAACTCC–TGGGTGGAAAGTCAAACGAATGAAAAAATTAA
GAAGAAAGTCGAAAGAAGATTAACTCC–T––GTGGAAAGTCAAACGAATGAAAAAATTAA
c
SERPINB3 121 DAIKKFYQTSVESVDFANAPEESRKKINSWVESQTNEKIK...
HT3B3-KO DAIKKFYQTSVESVDFANAPEESRKKINSLGGKSNE Stop
SW756B3-KO DAIKKFYQTSVESVDFANAPEESRKKINSCGKSNE Stop
d
rSERPINB3
rSERPINB4
HT3B3-WT
HT3B3-KO
SW756B3-WT
SW756B3-KO
75
50
37
α-SERPINB3/4
75
50
α-SERPINB4
50
37
α-actin
e
**
*
2-ΔΔCt
1.2
1.0
0.8
0.6
0.4
0.2
0.0
HT3B3-WT
HT3B3-KO
SW756B3-WT
SW756B3-KO

Supplementary Figure 5. CRISPR/Cas9 engineered tumor epithelial cell lines are null for SERPINB3. (a) Schematic representation of the genomic structure of human SERPINB3 with the gRNA target sequence shown between residues 432 and 451 within Exon 5 with the PAM site in red letters. (b) Sequence alignment of the wild-type human SERPINB3 reference sequence (Accession number NM_006919.3) with the cDNA sequences amplified by RT-PCR from $HT3^{B3\text{-}KO}$ and $SW756^{B3\text{-}KO}$ total RNA isolation. The $HT3^{B3\text{-}KO}$ clone shows a single base pair (t) addition (red) whereas the $SW756^{B3\text{-}KO}$ clone shows a 2-base pair indel (fuscia), resulting in a frameshift prior to the reactive site loop. (c) ClustalW alignment of the predicted amino acid sequences of wild-type SERPINB3 (top line) and $HT3^{B3\text{-}KO}$ (middle line) and $SW756^{B3\text{-}KO}$ (bottom line) clones translated from amino acid 121. Both $HT3^{B3\text{-}KO}$ and $SW756^{B3\text{-}KO}$ clones show a premature stop codon at position 158 and 157, respectively. (d) Western blot analysis of protein levels of SERPINB3 in SW756 and HT3 control and B3 null cell lines using a monoclonal antibody (Novus) that recognizes both SERPINB3 and -B4 (α-SERPINB3/B4), 10C12 monoclonal antibody specific for SERPINB4 (α-SERPINB4) and actin (α-actin) control. Recombinant GST fusion SERPINB3 and -B4 (rhB3 and rhB4, respectively) serve as positive controls for the polyclonal antibody and both positive and negative controls for the monoclonal antibody. (e) Quantitative RT-PCR of SERPINB3 transcript levels (forward primer: CGCGGTCTCGTGCTATCTG, reverse primer: ATCCGAATCCTACTACAGCGG) for $HT3^{B3\text{-}WT}$ and $SW756^{B3\text{-}WT}$ (B3-WT) and the $HT3^{B3\text{-}KO}$ and $SW756^{B3\text{-}KO}$ clones (B3-KO) expressed in $2^{-\Delta\Delta Ct}$ normalized to the wild-type. Note the dramatically reduced levels of transcript in the $HT3^{B3\text{-}KO}$ cell line and the undetectable levels in the $SW756^{B3\text{-}KO}$ clones.

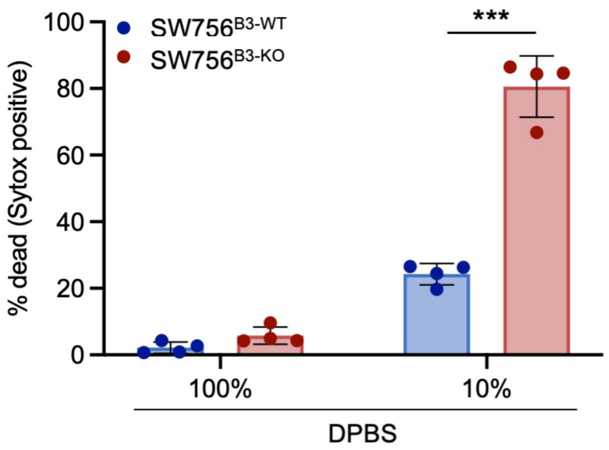
SW756B3-WT
SW756B3-KO
% dead (Sytox positive)
100
80
60
40
20
0
***
100%
10%
DPBS

Supplementary Figure 6. Loss of SERPINB3 also sensitizes SW756 cells to hypotonic stress. A representative experiment showing $SW756^{B3\text{-}WT}$ and $SW756^{B3\text{-}KO}$ cell lines treated with either 100% or 10% DPBS in the presence of Sytox Green for 4 hours. Cells were then stained with H33342 before imaging. The percent dead was calculated as (# of Sytox™ positive/# ofblue nuclei) × 100. The means ± SD were compared using a two-tailed *t*-test (***$P < 0.001$).

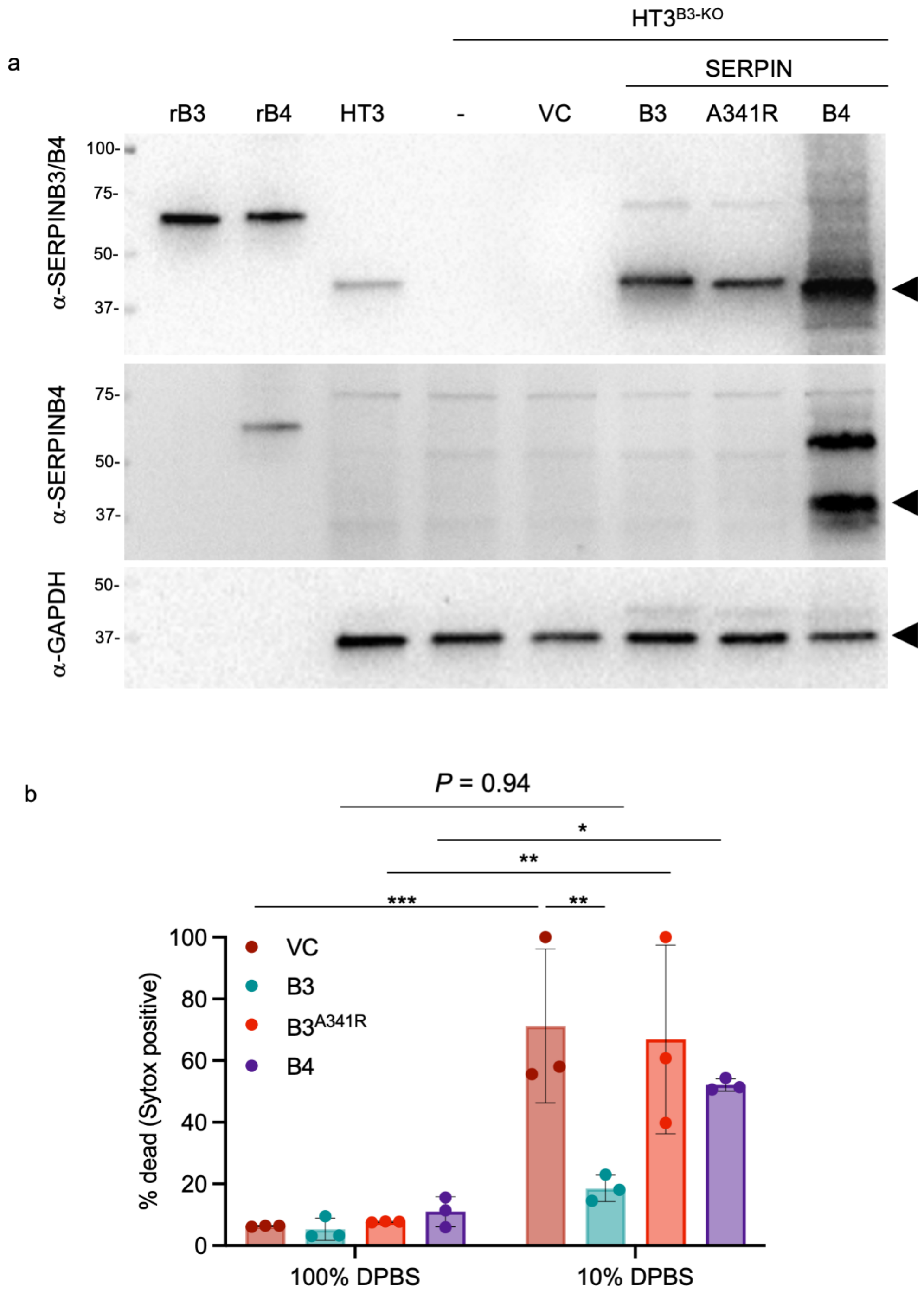
a
HT3B3-KO
SERPIN
rB3
rB4
HT3
-
VC
B3
A341R
B4
α-SERPINB3/B4
100-
75-
50-
37-
α-SERPINB4
75-
50-
37-
α-GAPDH
50-
37-
b
P = 0.94
*
**
***
**
100
80
60
40
20
0
% dead (Sytox positive)
VC
B3
B3A341R
B4
100% DPBS
10% DPBS

Supplementary Figure 7. SERPINB3 rescues HT3$^{B3\text{-}KO}$ cells from lysoptosis-like cell death. (a) Immunoblot analysis of cell lysates from HT3$^{B3\text{-}WT}$ and HT3$^{B3\text{-}KO}$ cells (-) nucleofected with empty vector (VC) or mammalian expression plasmids containing the cDNA for WT SERPINB3 (B3), SERPINB3^{A341R} (A341R) or SERPINB4 (B4) genes probed with a monoclonal antibody (Novus) that recognizes both SERPINB3 and SERPINB4 (α-SERPINB3/B4), 10C12 monoclonal antibody specific for SERPINB4 (α-SERPINB4) and actin (α-actin) control. Recombinant GST fusion SERPINB3 and -B4 (rB3 and rB4, respectively) serve as positive controls and both positive and negative controls for the monoclonal antibodies. (b) A representative experiment of the HT3 cells lines expressing empty vector control (VC; turquoise), SERPINB3 cDNA (B3; teal), the SERPINB3^{A341R} inactivating mutation cDNA (B3^{A341R}; red) and the SERPINB4 cDNA (B4; purple). Error bars represent the standard deviation from the mean. Statistical significance was analysed using a 2-way ANOVA with Tukey multiple comparisons (* $P < 0.05$; ** $P < 0.01$, *** $P < 0.001$). Note, there is no statistical difference between the 100% DPBS and 10% DPBS treated HT3$^{B3\text{-}KO}$ expressing SERPINB3 ($P = 0.94$).

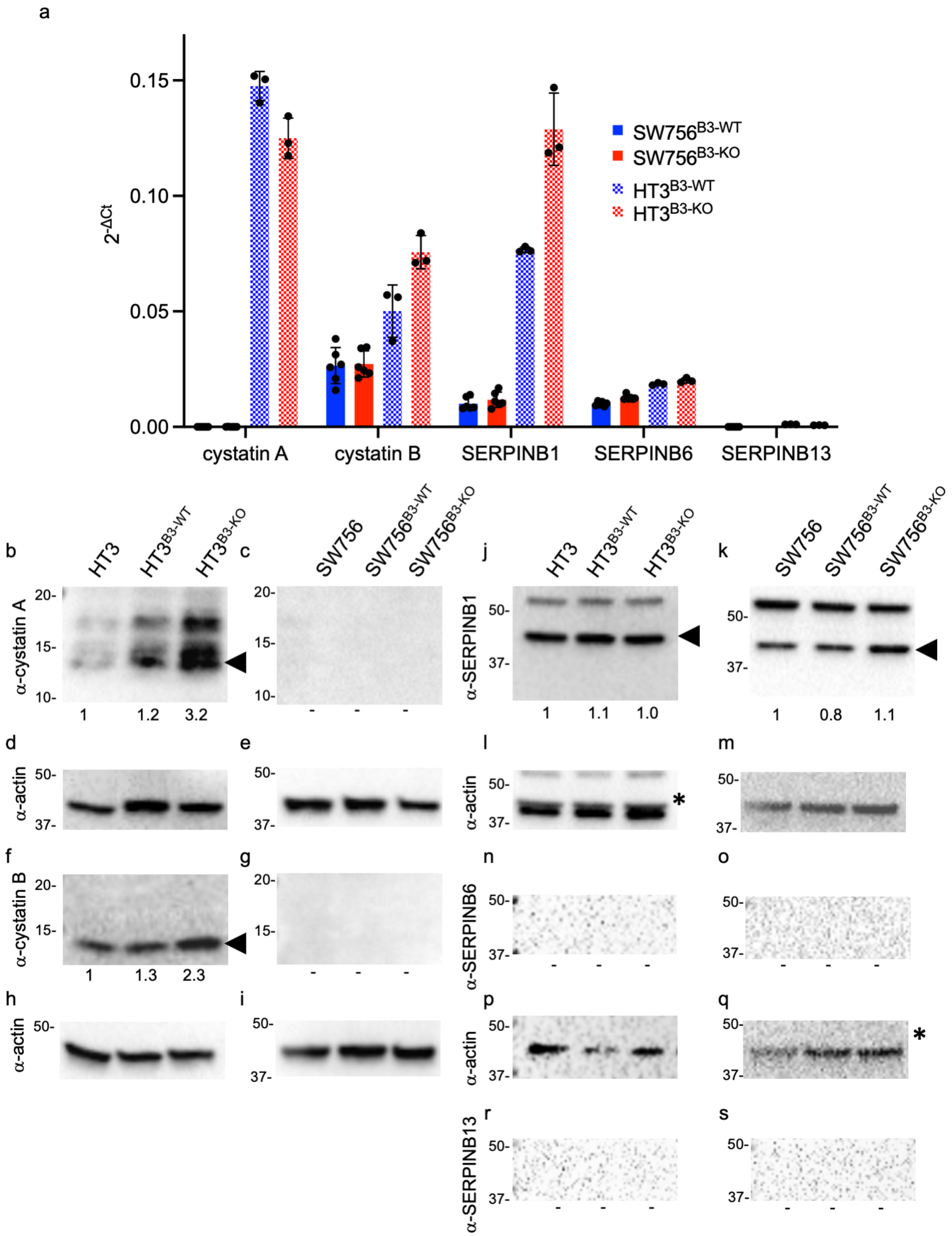

a
0.15
0.10
0.05
0.00
2^-ΔCt
SW756^B3-WT
SW756^B3-KO
HT3^B3-WT
HT3^B3-KO
cystatin A
cystatin B
SERPINB1
SERPINB6
SERPINB13
b
HT3
HT3^B3-WT
HT3^B3-KO
α-cystatin A
1
1.2
3.2
c
SW756
SW756^B3-WT
SW756^B3-KO
j
α-SERPINB1
1
1.1
1.0
k
1
0.8
1.1
d
α-actin
e
l
m
f
α-cystatin B
1
1.3
2.3
g
n
α-SERPINB6
o
h
i
p
q
r
α-SERPINB13
s

Supplementary Figure 8. Analysis of the expression of other functionally relevant protease inhibitors in SW756 and HT3 cell lines. Quantitative (q) RT-PCR (a) and western blot (WB) analysis (b-s) of cystatin A (qRT-PCR forward primer: GTACTTACTGGATACCAGGTTGAC, reverse primer: AGTAGCCAGTTGAAGGAATCAG; WB primary antibody clone B-11, 1 in 1000 dilution Santa Cruz, sc-376759), cystatin B (qRT-PCR forward primer: GTACTTACTGGATACCAGGTTGAC, reverse primer: AGTAGCCAGTTGAAGGAATCAG; WB primary antibody clone F-5, 1 in 1000 dilution Santa Cruz, sc-166561), SERPINB1 (qRT-PCR forward primer: AGGTGTGCAGGATCTCTTTAAC, reverse primer: CCTCTGTTCCCTCTTCATTCAC WB primary antibody clone 4D7, 1 in 1000 dilution, Santa Cruz, sc-293462), SERPINB6 (qRT-PCR forward primer: GGTTCTGGTGAATGCTGTCTAT, reverse primer: GCACAGGTTTCTCCTCATTCT; WB primary antibody clone E-8, 1 in 1000 dilution Santa Cruz, sc-398487) and SERPINB13 (qRT-PCR forward primer: CGATGGCCTGGAGAAGATAATAG, reverse primer: CAAGTGCAGATTCACCTTTCTTT; primary antibody clone B29, 1 in 1000 dilution Millipore-Sigma, MABS1320). Transcript levels for $HT3^{B3\text{-}WT}$ and $SW756^{B3\text{-}WT}$ and the $HT3^{B3\text{-}KO}$ and $SW756^{B3\text{-}KO}$ clones expressed in $2^{-\Delta Ct}$. Relative intensities compared to actin-HRP (1 in 1000 dilution) for WB are located below gel. Asterix's indicate actin blots that were stripped and reprobed for SERPINB13. Note, some of the actin blots are duplicated Fig S12 as they were stripped and reprobed for cathepsin primary antibodies.

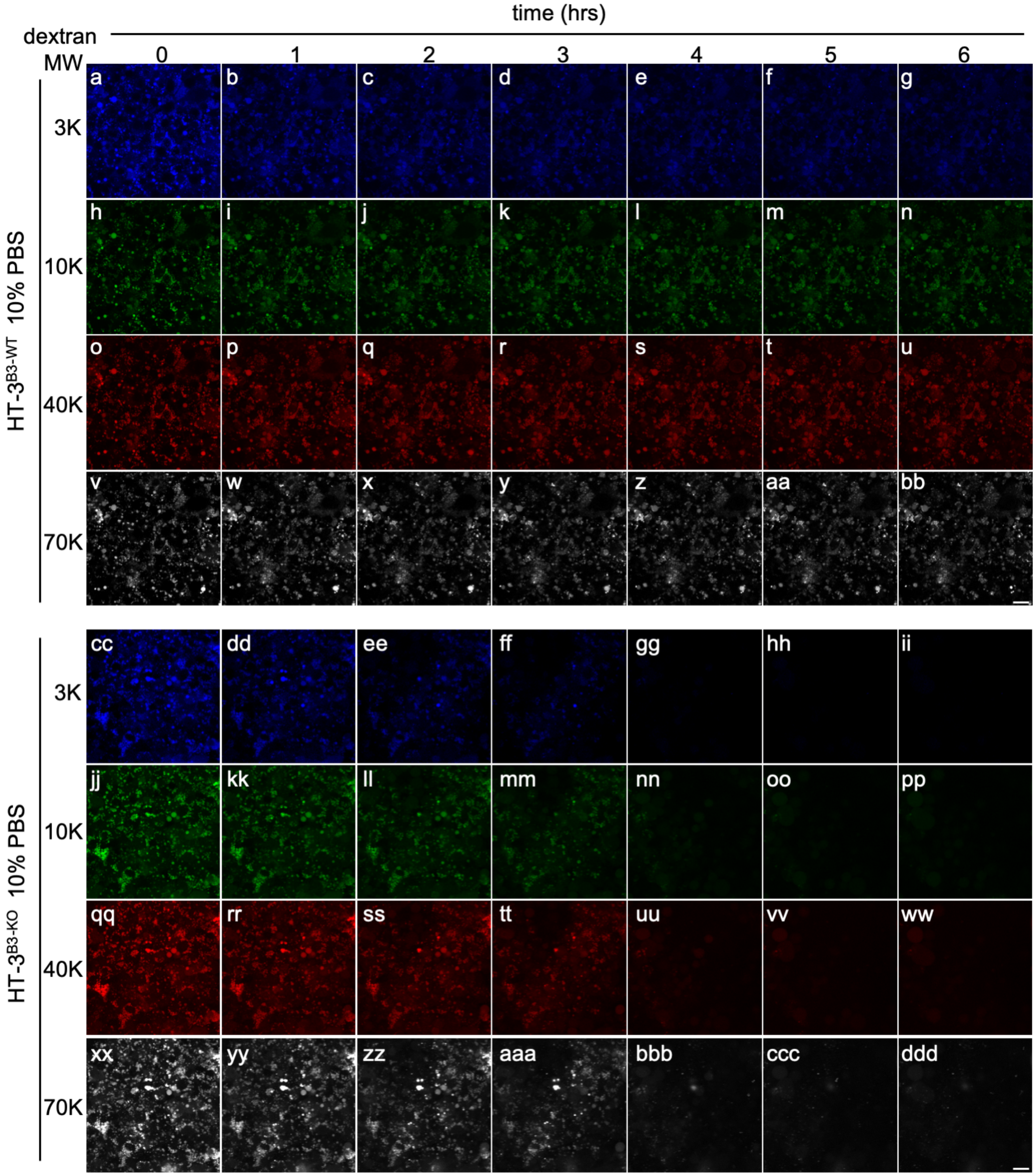

time (hrs)
dextran MW
0
1
2
3
4
5
6
HT-3B3-WT 10% PBS
3K
10K
40K
70K
a b c d e f g
h i j k l m n
o p q r s t u
v w x y z aa bb
HT-3B3-KO 10% PBS
cc dd ee ff gg hh ii
jj kk ll mm nn oo pp
qq rr ss tt uu vv ww
xx yy zz aaa bbb ccc ddd

Supplementary Figure 9. Examples of quadruple fluorescent labeled lysosome imaging using for quantification in HT3$^{B3\text{-}WT}$ and HT3$^{B3\text{-}KO}$ cell lines treated with hypotonic stress. (a-ddd) Representative maximum intensity projections of HT3$^{B3\text{-}WT}$ (a-bb) and HT3$^{B3\text{-}KO}$ (cc-ddd) cell lines stained with 3 kDa Cascade blue (3K; blue; a-g; cc-ii), 10 kDa Alexa488 (10K; green; h-n; jj-pp), 40 kDa TMR (40K; red; o-u; qq-ww) and 70 kDa Texas red (70K; white; v-bb; xx-ddd) conjugated dextrans prior to exposure to 10% DPBS. Cells were imaged using live cell resonance scanning confocal microscopy in x, y and z (≥ 20 z-planes) over 6 hours at 37 ºC and 5% CO_2.

a

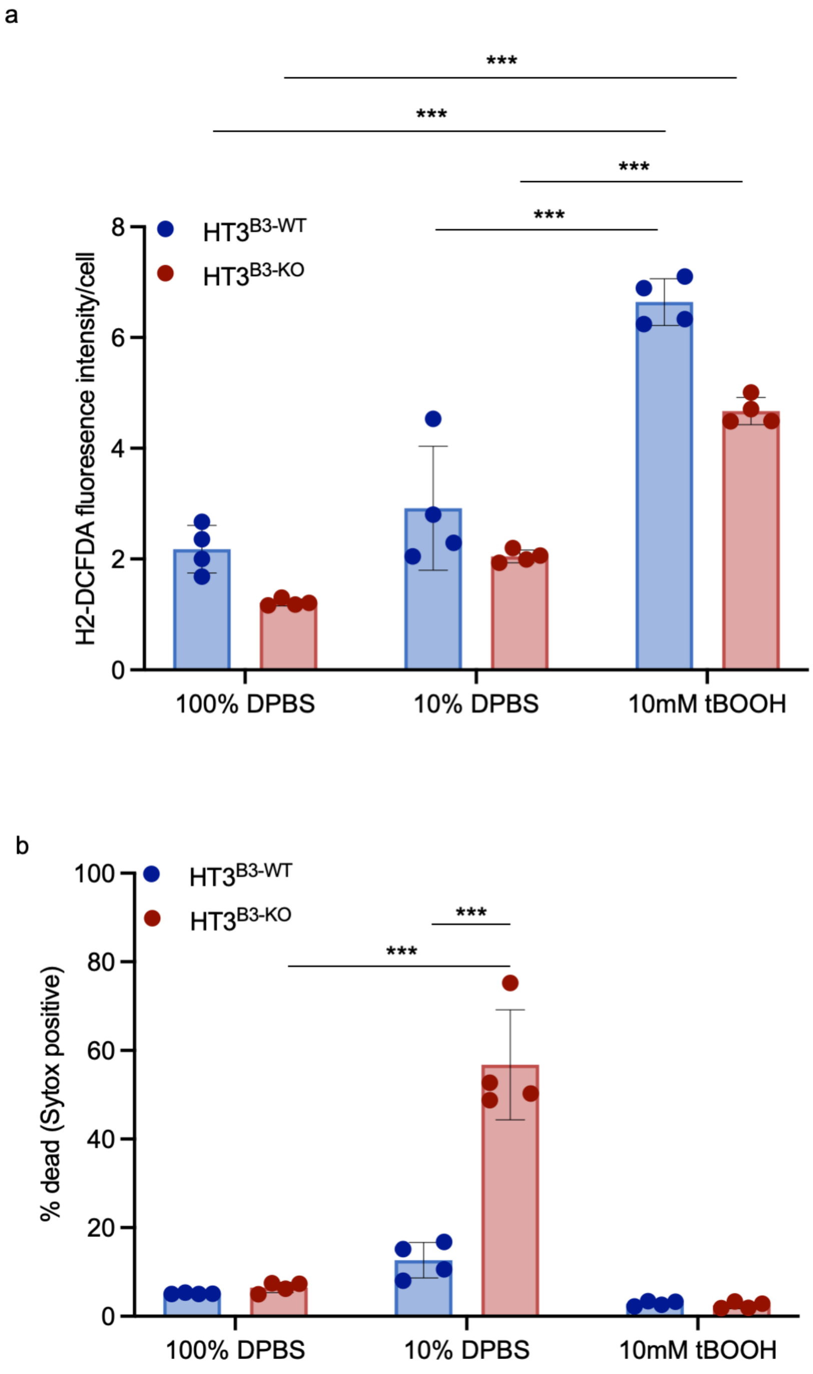

a
***
***
***
***
HT3B3-WT
HT3B3-KO
H2-DCFDA fluoresence intensity/cell
8
6
4
2
0
100% DPBS
10% DPBS
10mM tBOOH
b
100
80
60
40
20
0
% dead (Sytox positive)
***
***
HT3B3-WT
HT3B3-KO
100% DPBS
10% DPBS
10mM tBOOH

Supplementary Figure 10. Hypotonic stress does not induce ROS in HT3^{B3-WT} or HT3^{B3-KO} cells. A representative experiment of HT3^{B3-WT} and HT3^{B3-KO} cells lines treated with either 100% DPBS (negative control), 10% DPBS or 10 mM *tert*-butylhydroperoxide (tBOOH) (ROS indicator positive control) in the presence of the ROS indicator H2-DCFDA and Sytox orange and imaged using the Cytation 5 at 4 hours at the appropriate wavelengths at 37 ºC and 5% CO_2. The total number of cells was calculated using the high contrast brightfield channel. The H2-DCFDA fluorescence intensity was normalized by dividing by the total number of cells (a) and the percent dead was calculated by the number of sytox positive cells/total number of cells $\times$ 100 (b). Significance was determined using a two-way ANOVA with Tukeys' multiple comparisons. Note, the significant increase in H2-DCFDA fluorescence intensity in the 10 mM tBOOH positive controls vs the 100% and 10% DPBS treatment.

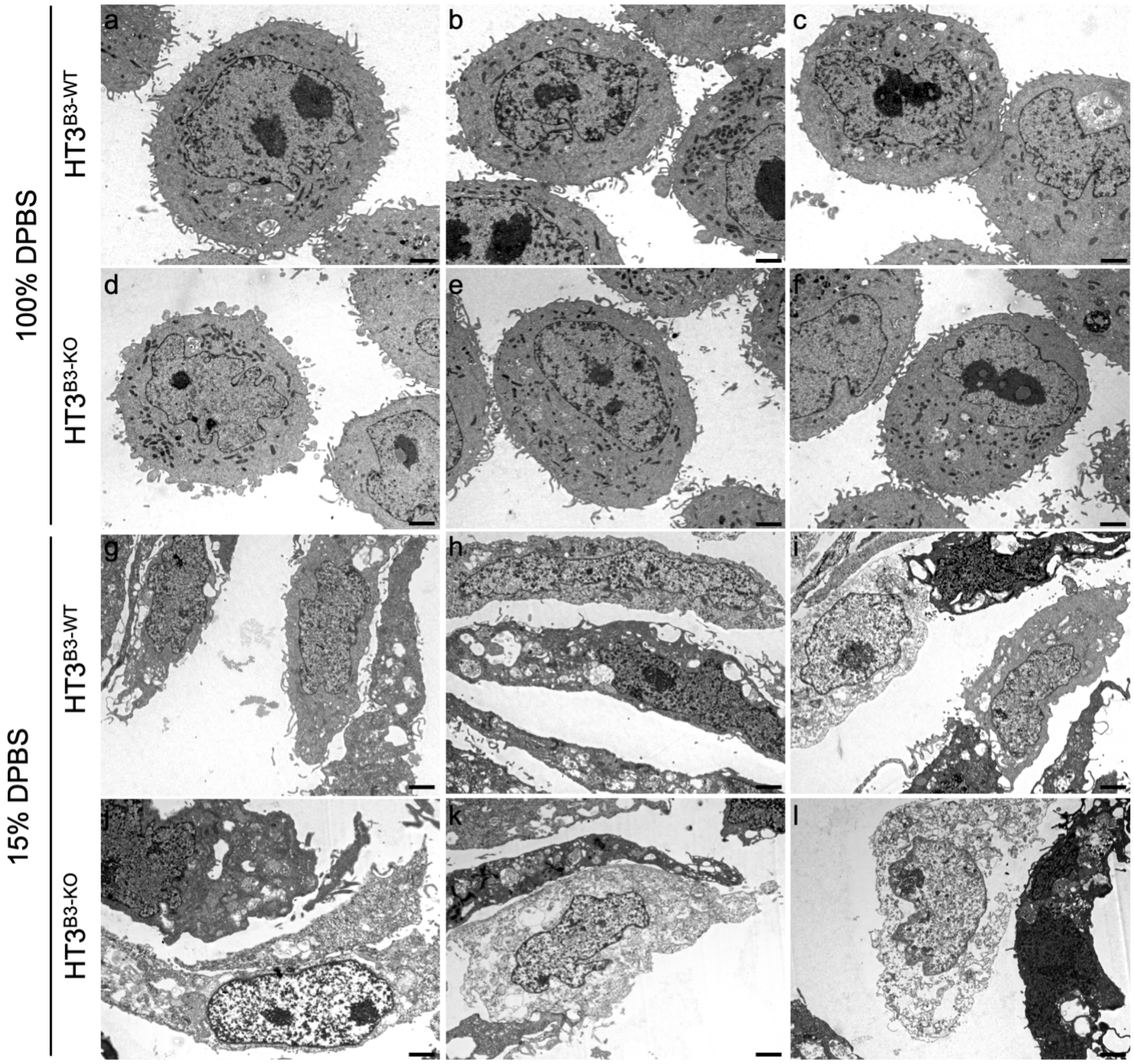
100% DPBS
15% DPBS
HT3B3-WT
HT3B3-KO
HT3B3-WT
HT3B3-KO
a
b
c
d
e
f
g
h
i
j
k
l

Supplementary Figure 11. Additional transmission electron micrographs from multiple fields of view of HT3 cells treated with hypotonic stress. (a-l) Additional low power (2000 x magnification) TEM images (scale bars = 2 μm) of either $HT3^{B3\text{-}WT}$ (a-c; g-i) or $HT3^{B3\text{-}KO}$ (d-f; j-l) cells treated with either 100% (a-f) or 15% DPBS (g-l) for 4 hours. Note the higher number of cells displaying morphological necrotic cell death characteristics is greater in $HT3^{B3\text{-}KO}$ cells treated with 15% DPBS.

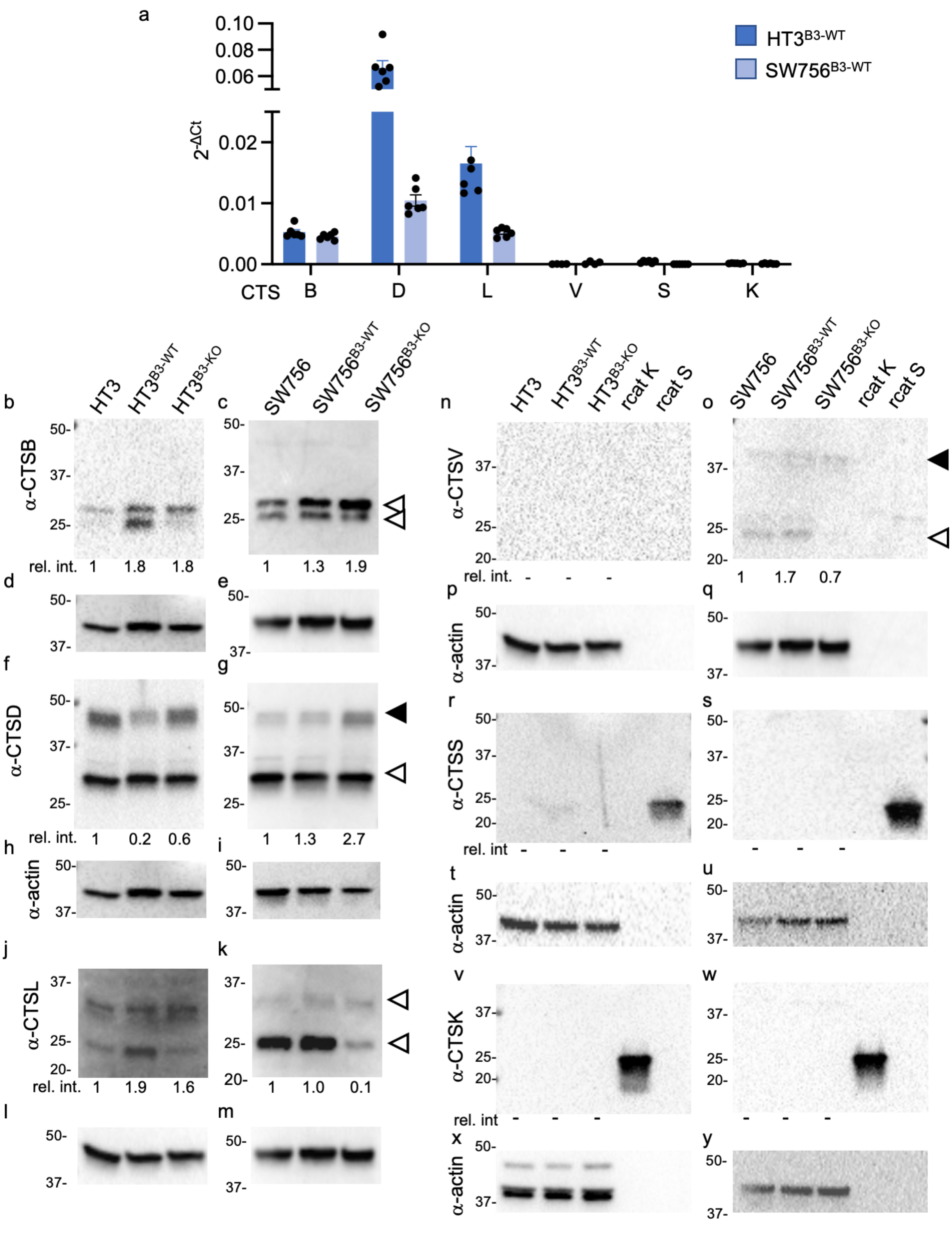
a
HT3B3-WT
SW756B3-WT
0.10
0.08
0.06
0.02
0.01
0.00
2-ΔCt
CTS B D L V S K
b c n o
HT3 HT3B3-WT HT3B3-KO
SW756 SW756B3-WT SW756B3-KO
HT3 HT3B3-WT HT3B3-KO rcat K rcat S
SW756 SW756B3-WT SW756B3-KO rcat K rcat S
α-CTSB
α-CTSV
α-CTSD
α-CTSL
α-CTSS
α-CTSK
α-actin
rel. int. 1 1.8 1.8
1 1.3 1.9
rel. int. - - -
1 1.7 0.7
d e p q
f g r s
rel. int. 1 0.2 0.6
1 1.3 2.7
rel. int - - -
- - -
h i t u
j k v w
rel. int. 1 1.9 1.6
1 1.0 0.1
rel. int - - -
- - -
l m x y
50-
37-
25-
20-

Supplementary Figure 12. HT3 and SW756 cells lines predominantly contain cathepsins B, -D and –L. (a) Quantitative RT-PCR of cathepsin B (CTSB; qRT-PCRforward primer: GAGCTGGTCAACTATGTCAACA, reverse primer: GCTCATGTCCACGTTGTAGAAGT), cathepsin D (qRT-PCR forward primer: TGCTCAAGAACTACAATGGACGC, reverse primer: CGAAGACGACTGTGAAGCACT), cathepsin S (CTSS; qRT-PCR forward primer: AAACGGCTGGTTTGTGTGC, reverse primer: CAGTGGTGATCCAGGGTAGG), cathepsin K (CTSK; qRT-PCR forward primer: ACACCCACTGGGAGCTATG, reverse primer: GACAGGGGTACTTTGAGTCCA), cathepsin L (CTSL; qRT-PCR forward primer: CTTTTGCCTGGGAATTGC, reverse primer: CATCGCCTTCCACTTGGTC) and cathepsin V (CTSV; qRT-PCR forward primer: GGAAGTAAGGAAGGGTGAGATAG, reverse primer: CCTCTAGTATGATGCCCAAGTG), cathepsin S (CTSS; qRT-PCR forward primer: AAACGGCTGGTTTGTGTGC, reverse primer: CAGTGGTGATCCAGGGTAGG), cathepsin K (CTSK; qRT-PCR forward primer: ACACCCACTGGGAGCTATG, reverse primer: GACAGGGGTACTTTGAGTCCA). Transcript levels for $HT3^{B3\text{-}WT}$ and $SW756^{B3\text{-}WT}$ clones expressed in $2^{-\Delta Ct}$. (b-y) Western blot analysis of the levels of CTSB (b, c), CTSD (f, g), CTSL (j, k), CTSV (n, o), CTSS (r, s) and CTSK (v, w) for HT3 parental cell line (HT3), $HT3^{B3\text{-}WT}$, $HT3^{B3\text{-}KO}$ (b, f, j, n, r, & v), SW756 parental cell line (SW756), $SW756^{B3\text{-}WT}$ and $SW756^{B3\text{-}KO}$ (c, g, k, o, s & w). Black arrowheads denote inactive pro-domain containing forms of the proteases and open arrow denote active forms. Relative intensities (rel. int.) compared to actin-HRP and normalized to the corresponding parental cell line (d e, h, i, l, m, p, q, t, u, x & y) for WB are located below gel. Note, the following actin blots are for the same lysates from blots that was stripped and reprobed:

Figs S8d, S12d and S12h; Figs S8h, S12l and S12p; Figs S8l and S12x; S8i, S12e, S12m and S12q; Figs S8q and S12u; Figs S8m and S12y. These blots were duplicated below their corresponding blots for clarity.

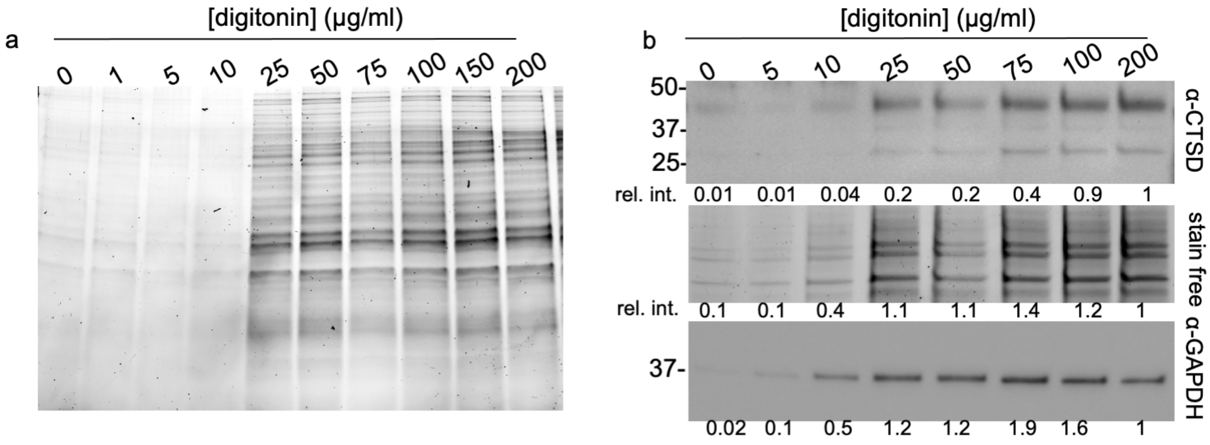
a
[digitonin] (µg/ml)
0 1 5 10 25 50 75 100 150 200
b
[digitonin] (µg/ml)
0 5 10 25 50 75 100 200
50-
37-
25-
α-CTSD
rel. int. 0.01 0.01 0.04 0.2 0.2 0.4 0.9 1
stain free
rel. int. 0.1 0.1 0.4 1.1 1.1 1.4 1.2 1
37-
α-GAPDH
0.02 0.1 0.5 1.2 1.2 1.9 1.6 1

Supplementary Figure 13. Digitonin can specifically release cytoplasmic contents at low concentrations in HT3 cell line. Determination of digitonin concentration that releases cytosolic content without disrupting lysosomes. (a) Stain-free 4-16% gradient SDS-PAGE (Bio-Rad) of supernatents from $HT3^{B3\text{-}KO}$ cells treated with the indicated concentration of digitonin for 30 minutes at 4ºC in DPBS. Note the increase in total protein release at ≥25 µg/ml digitonin concentration. (b) Western blot analysis of lysosomal CTSD and cytosolic GAPDH with corresponding stain free SDS-PAGE of supernatents from $HT3^{B3\text{-}KO}$ cells treated with the indicated concentration of digitonin for 30 minutes at 4ºC. The background subtracted relative band intensities (rel. int.) for each lane were calculated (Image Lab, v6.1, Bio-Rad) and normalized to the highest concentration of digitonin (200 µg/ml).

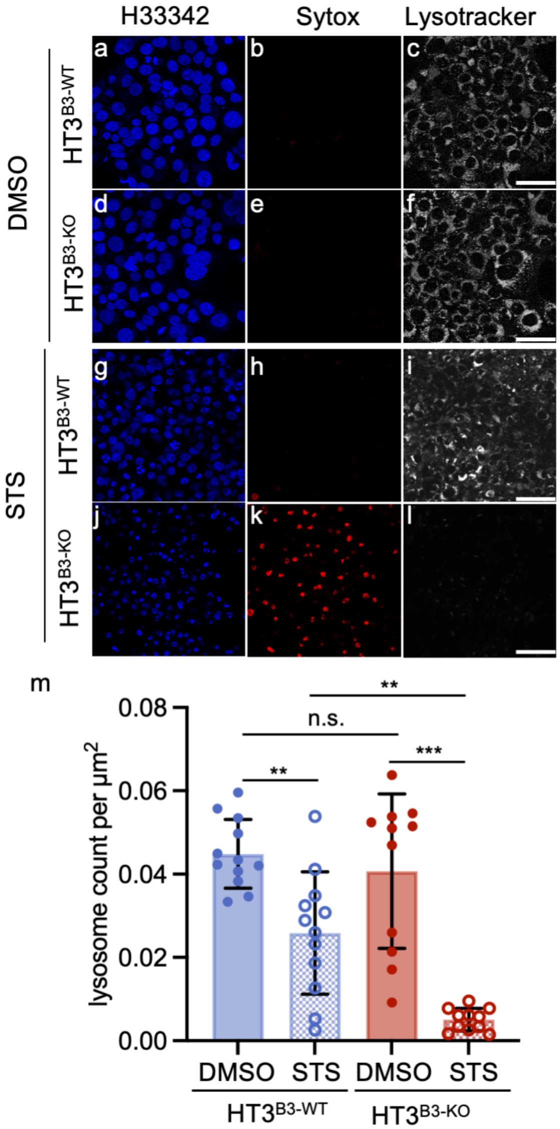
H33342
Sytox
Lysotracker
a
b
c
d
e
f
g
h
i
j
k
l
DMSO
STS
HT3B3-WT
HT3B3-KO
HT3B3-WT
HT3B3-KO
m
lysosome count per µm2
0.08
0.06
0.04
0.02
0.00
n.s.
**
**
***
DMSO
STS
DMSO
STS
HT3B3-WT
HT3B3-KO

Supplementary Figure 14. HT3$^{B3\text{-}KO}$ cells lose Lysotracker™ staining after exposure to staurosporine. (a-l) Representative confocal images of HT3$^{B3\text{-}WT}$ and HT3$^{B3\text{-}KO}$ cells treated with DMSO control or 5 µM STS for 16 hours and stained with H33342 (blue), Sytox Orange (red) and lysotracker deep red (white). Scale bars represent 25 µm. (m) Quantification of the lysotracker positive vesicles (lysosomal count) per µm^2 of cellular area in confocal images (shown in panels a-l) of HT3$^{B3\text{-}WT}$ (blue bars) or HT3$^{B3\text{-}KO}$ (red bars) cell lines were treated with either DMSO (solid fill) or STS (patterned fill) over multiple fields of view (n≥10). Analyses were compared using an ordinary one way ANOVA with Tukeys' multiple comparisons (** $P < 0.01$, *** $P < 0.001$).

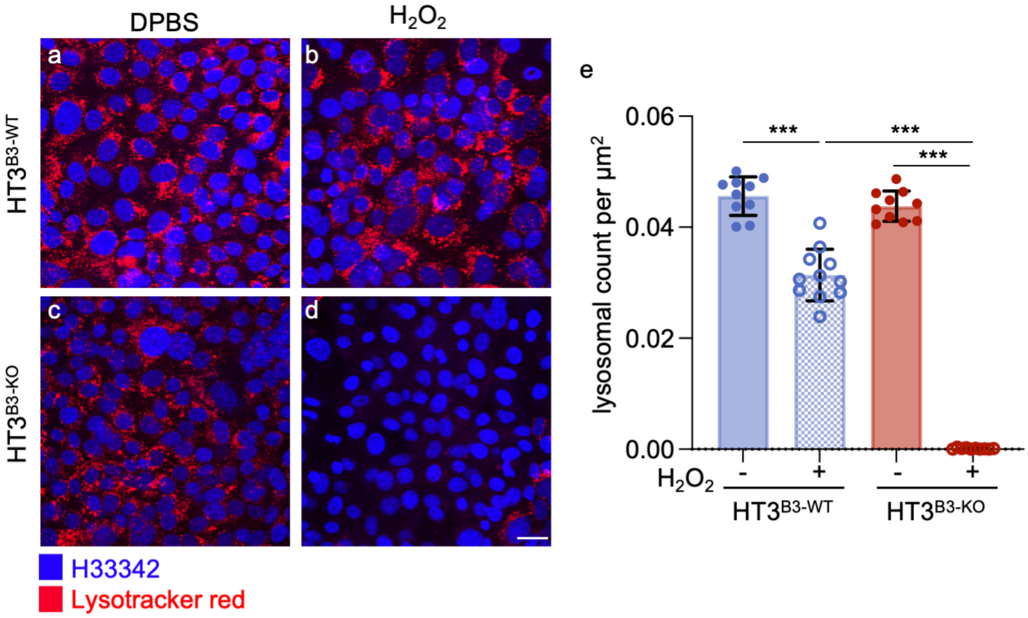
DPBS
H_2O_2
a
b
c
d
e
$HT3^{B3-WT}$
$HT3^{B3-KO}$
H33342
Lysotracker red
lysosomal count per μm^2
0.06
0.04
0.02
0.00
***
***
***
H_2O_2
-
+
-
+
$HT3^{B3-WT}$
$HT3^{B3-KO}$

Supplementary Figure 15. HT3$^{B3\text{-}KO}$ cells have decreased Lysotracker red staining with H_2O_2 treatment. (a-d) Representative maximum intensity projections of either HT3$^{B3\text{-}WT}$ (a-b) or HT3$^{B3\text{-}KO}$ (c-d) cells treated with 5 mM H_2O_2 for 8 hours. Cells were stained with H33342 (blue) and Lysotracker Red (red) prior to imaging over x, y and z at multiple fields of view (n ≥ 10) using confocal microscopy. Scale bar represents 25 µm. Note the almost complete loss of lysosomal staining observed in the HT3$^{B3\text{-}KO}$ cells treated with H_2O_2 (d). (e) Quantification of the lysotracker positive vesicles (lysosomal count) per µm^2 of cellular area in HT3$^{B3\text{-}WT}$ (blue bars) or HT3$^{B3\text{-}KO}$ (red bars) cells treated with H_2O_2 (+; patterned fill) or the equivalent volume of DPBS (-; solid fill) over multiple fields of view (n ≥ 10, *** $P < 0.001$, two-tailed *t*-Test).

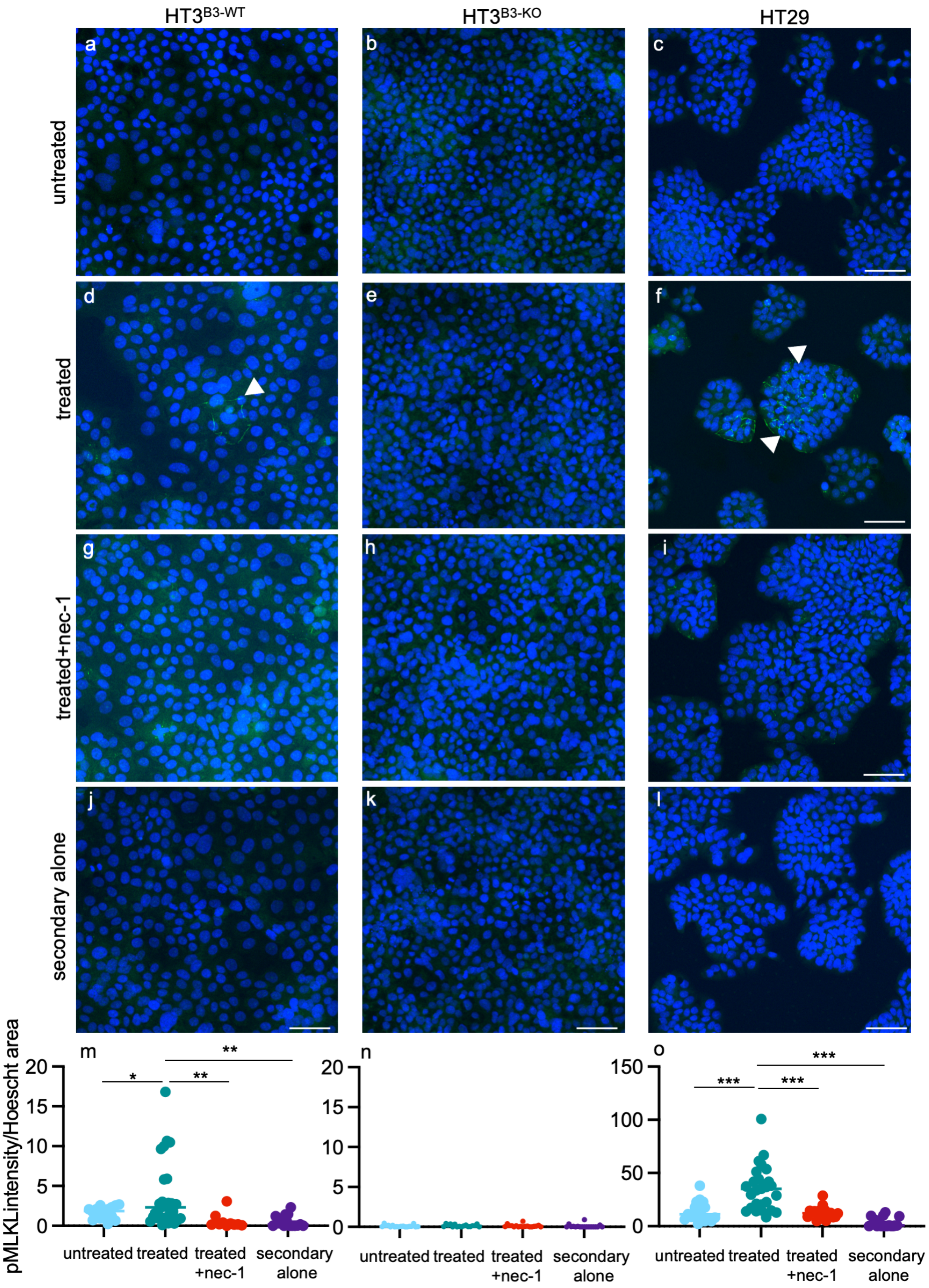

HT3B3-WT
HT3B3-KO
HT29
a
b
c
d
e
f
g
h
i
j
k
l
untreated
treated
treated+nec-1
secondary alone
m
n
o
pMLKLintensity/Hoescht area
*
**
***
untreated
treated
treated +nec-1
secondary alone

Supplementary Figure 16. pMLKL can be induced in HT3$^{B3\text{-}WT}$ cells treated with necroptosis inducers. (a-l) Representative confocal images of HT3$^{B3\text{-}WT}$ (a, d, g, j), HT3$^{B3\text{-}KO}$ (b, e, h, k) and HT29 (positive control; c, f, i, l) cell lines treated with diluent (untreated; a-c) TNFα + BV6 + qVD-OPh + cyclohexamide (HT3 cells) or TNFα + zVAD-fmk (HT29) (treated; d-f), the same treatments but in the presence of 10 μM necrostatin-1 (treated+nec-1, g-i) for 8 hours prior to fixation and immunofluorescence detection of phospho-MLKL (green) and counterstained with Hoescht 33342 (blue). As a background fluoresence control, the primary phospho-MLKL was omitted from staining in a subset of untreated cells (secondary alone, j-l). White arrowheads indicate areas of phosphor-MLKL plasma membrane staining. (m-o) Quantification of multiple fields of the phospho-MLKL staining fluorescence above background normalized to the number of cells imaged as indicated by the total Hoescht 33342 area (pMLKL instenisty/Hoescht area) over n≥10 separate fields per treatment. Statistical significance was determined using a two-way ANOVA with Tukeys' multiple comparisons (* $P < 0.05$, ** $P < 0.01$, *** $P < 0.001$). Note, the statistically significant increase in pMLKL instenisty/Hoescht area between HT3$^{B3\text{-}WT}$ and HT29 untreated and treated samples that was abrogated with nec-1 treatment.

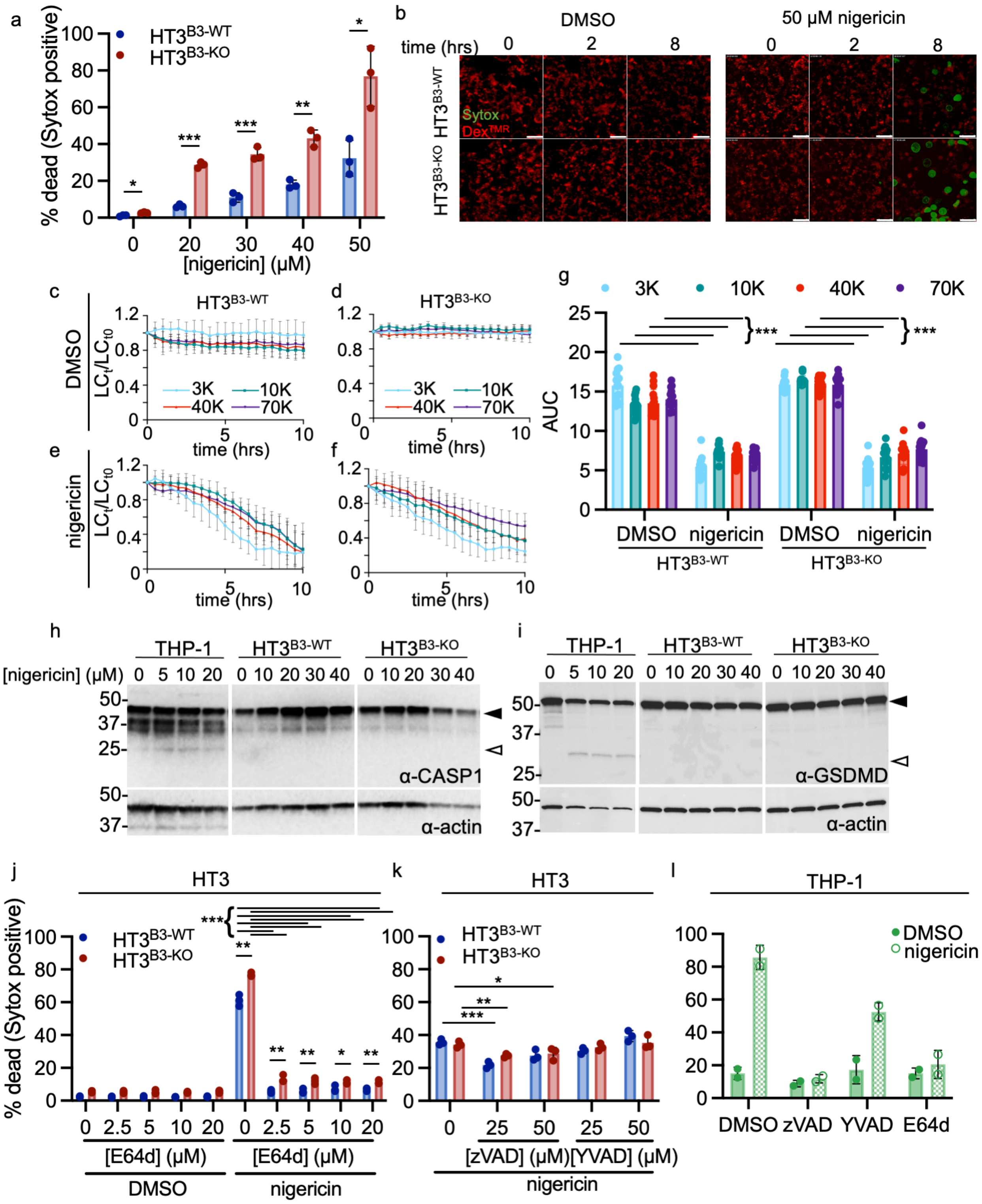

a
% dead (Sytox positive)
HT3B3-WT
HT3B3-KO
[nigericin] (μM)
b
DMSO
50 μM nigericin
time (hrs)
Sytox
DexTMR
c
d
e
f
DMSO
nigericin
LCt/LCt0
time (hrs)
3K
10K
40K
70K
g
AUC
h
i
THP-1
[nigericin] (μM)
α-CASP1
α-GSDMD
α-actin
j
k
l
HT3
[E64d] (μM)
[zVAD] (μM)
[YVAD] (μM)
nigericin
DMSO
zVAD
YVAD
E64d

Supplementary Figure 17. Nigericin does not induce pyroptosis in HT3 cells but does induce LMP. (a) Either $HT3^{B3\text{-}WT}$ (blue bars) or $HT3^{B3\text{-}KO}$ (red bars) cells were incubated with the indicated concentration of nigericin for 24 hours at 37ºC / 5% CO_2 in the presence of Sytox Orange. Cells were stained with H33342 and the percent dead was calculated as (# Sytox positive nuclei/# of blue nuclei) × 100. The means ± SD of a representative experiment were compared using a two-tailed *t*-test (****P*< 0.001, ***P*<0.01, * *P*<0.05). Note that at all concentrations of DMSO there is a statistical increase in % dead in the $HT3^{B3\text{-}KO}$ cells. (b) Representative maximum intensity projections of either $HT3^{B3\text{-}WT}$ or $HT3^{B3\text{-}KO}$ labeled with 10kDa TMR-dextran (red) and exposed to DMSO diluent control or 50 µM nigericin in the presence of Sytox Green (green). Cells were imaged by live-cell time lapse confocal microscopy in X, Y, Z at multiple fields of view. The indicated time points are shown. Scale bars represent 25 µm. Note the loss of lysosomal staining in both cell lines but an increase in Sytox Green staining in $HT3^{B3\text{-}KO}$. (c-f) To quantify lysosomal content, $HT3^{B3\text{-}WT}$ or $HT3^{B3\text{-}KO}$ cells were incubated with fluorescently labeled different molecular weight dextrans as previously stated, treated with either diluent control (DMSO) or 50 µM nigericin at multiple positions (fields ≥ 10) over 10 hours at 37ºC and 5% CO_2 and imaged using live-cell confocal microscopy. Number of lysosomes at each time point (LC_t) were normalized to the lysosome count at time zero (LC_{t0}). (g) AUC for each dextran over time for experiment in c-f (compared by 2-way ANOVA with Tukey's multiple comparisons test). (h-i) Western blot analysis of caspase-1 (CASP1) or gasdermin D (GSDMD) cleavage and actin protein levels from LPS pretreated THP-1, $HT3^{B3\text{-}WT}$ and $HT3^{B3\text{-}KO}$ cell lysates after exposure to the indicated concentrations of

nigericin for 5 hours. Note the presence of P25 and P30 cleavage band (open arrowhead) in the THP-1 cell line for CASP1 and GSDMD, respectively. There was no detectable cleavage of CASP1 or GSDMD in the HT3 cell lines. (j, k) Either $HT3^{B3\text{-}WT}$ (blue bars) or $HT3^{B3\text{-}KO}$ (red bars) cells were incubated with the indicated concentration of E64d (j), z-VAD-fmk and YVAD-CHO (k) for 1 hour at 37ºC / 5% CO_2 in the presence of Sytox Orange. Cells were then incubated with diluent control (DMSO) or 50 µM nigericin for 16 hours and stained with H33342. The percent dead was calculated as (# Sytox positive nuclei/# of blue nuclei) × 100. The means ± SD of a representative experiment were compared using a two-tailed *t*-test (****P*< 0.001, ***P*<0.01, * *P*<0.05). Notably, the protection in both HT3 cell lines treated with E64d with little protection with z-VAD-fmk and no protection of YVAD-CHO. (l) THP-1 (green bars) cells were incubated with 1 µg / ml LPS and 50 µM of the indicated inhibitor for 3 hours at 37ºC / 5% CO_2 Cells were then treated with DMSO diluent control (solid fill) or 10 µM nigericin (pattern fill) for 2 hours in the presence of Sytox Orange. Prior to imaging, cells were stained with H33342 and the percent dead was calculated as (# Sytox positive nuclei/# of blue nuclei) × 100. The means (n = 2) ± SD of a representative experiment are shown. Notably, the protection from Sytox staining was observed with z-VAD-fmk, YVAD-CHO and E64d.

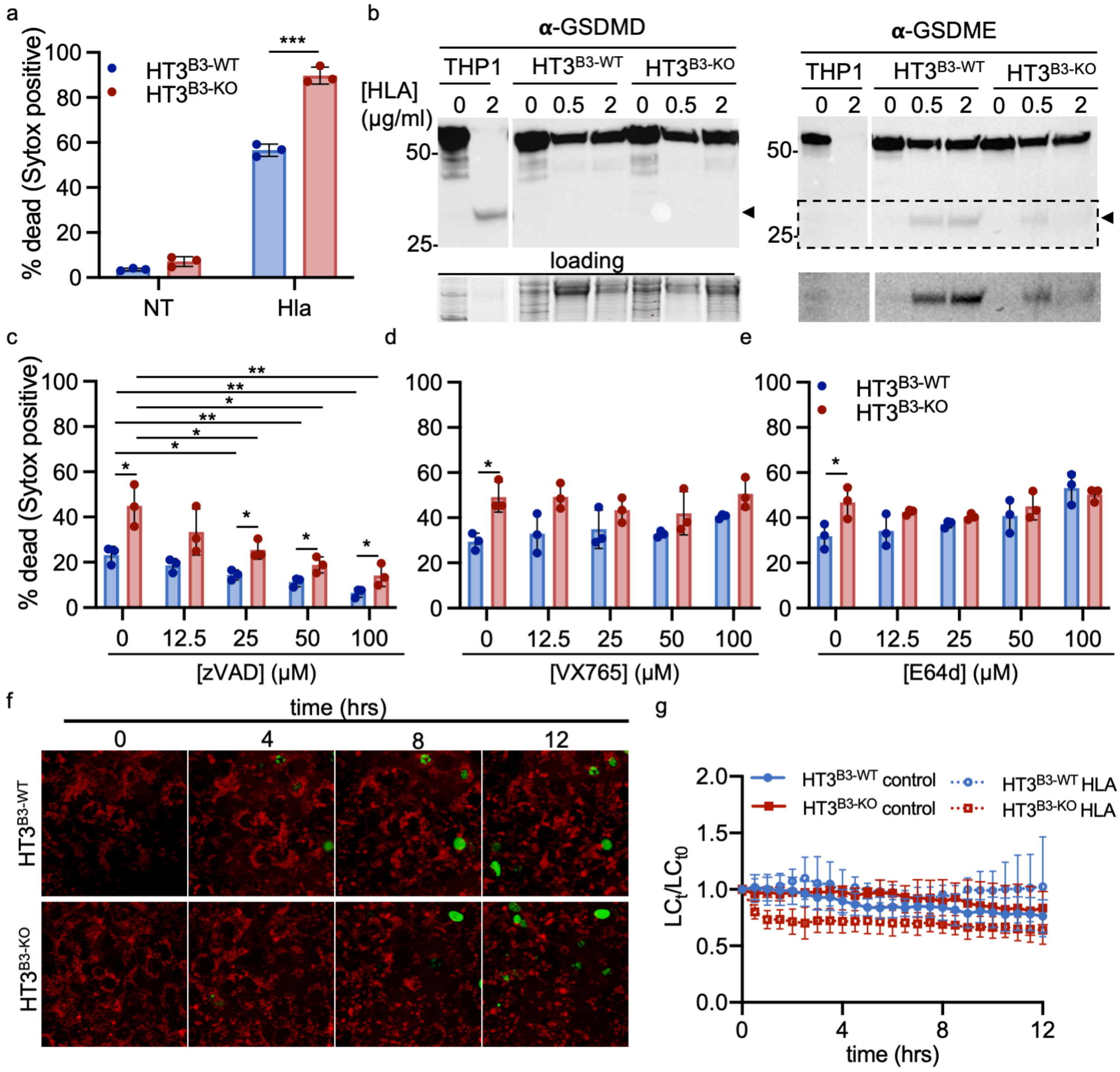
a
% dead (Sytox positive)
HT3B3-WT
HT3B3-KO
***
NT
Hla
b
α-GSDMD
THP1
HT3B3-WT
HT3B3-KO
[HLA] (μg/ml)
0 2 0 0.5 2 0 0.5 2
50-
25-
loading
α-GSDME
25
c
d
e
[zVAD] (μM)
[VX765] (μM)
[E64d] (μM)
0 12.5 25 50 100
f
time (hrs)
0 4 8 12
g
HT3B3-WT control
HT3B3-KO control
HT3B3-WT HLA
HT3B3-KO HLA
LCt/LCt0
time (hrs)

Supplementary Figure 18. Alpha hemalysin induces gasdermin E cleavage in HT3 cells. (a) Either HT3$^{B3\text{-}WT}$ (blue bars) or HT3$^{B3\text{-}KO}$ (red bars) cells were incubated without (NT) or with 1 µg / ml of alpha hemalysin (Hla) for 24 hours at 37ºC / 5% CO_2 in the presence of Sytox Orange. Cells were stained with H33342 and the percent dead was calculated as (# Sytox positive nuclei/# of blue nuclei) × 100. The means ± SD of a representative experiment were compared using a two-tailed *t*-test (****P*<0.001). (b) Western blot analysis of gasdermins D (GSDMD) and E (GSDME) in THP-1, HT3$^{B3\text{-}WT}$ or HT3$^{B3\text{-}KO}$ cells treated with the indicated HLA concentration for 1.5 hours after LPS pretreatment for THP-1 cells and 16 hours for HT3 cell lines. Cell lysates were separated by SDS-PAGE and GSDMD and GSDME detected using the appropriate mAb. GSDMD and GSDME cleavage products are indicated by the black arrowheads. Hashed box in GSDME blot indicates the enhanced area below the gel. Numerals indicate the molecular weight markers in kDa. Total protein loading was indicated by stain free SDS-Page (BioRad). Note the presence of a GDSME band in HT3 cell lines absent in THP-1 cells and no detectable cleavage of GSDMD. (c-e) Either HT3$^{B3\text{-}WT}$ (blue bars) or HT3$^{B3\text{-}KO}$ (red bars) cells were incubated with the indicated concentration of z-VAD-fmk (c), caspase-1 inhibitor, VX735 (d) and E64d (e) for 3 hours prior to treatment with 1 µg / ml Hla at 37ºC / 5% CO_2 for 18 hours. Cells were stained with H33342 and the percent dead was calculated as (# Sytox positive nuclei/# of blue nuclei) × 100. The means ± SD of a representative experiment were compared using a two-tailed *t*-test (***P*<0.01, * *P<0.05*). Note that protection was only observed in z-VAD treated cells. (f) Representative maximum intensity projections of either HT3$^{B3\text{-}WT}$ or HT3$^{B3\text{-}KO}$ cells were labeled with 10 kDa TMR-

Dextran and imaged by time-lapse live cell confocal microscopy in X, Y, Z over multiple fields with 1 μg / ml Hla in the presence of Sytox Green over 12 hours. Note the lack of lysosomal loss in both HT3 cell lines. (g) Quantification of the lysosomal count at each time point of the images shown in F normalized to time 0. $HT3^{B3\text{-}WT}$ (blue lines, circles) or $HT3^{B3\text{-}KO}$ (red lines, squares) cells were not treated (control, filled symbols) or treated with 1 μg / ml Hla (dashed lines, open symbols) prior to imaging. Representative experiment over n ≥ 7 fields.

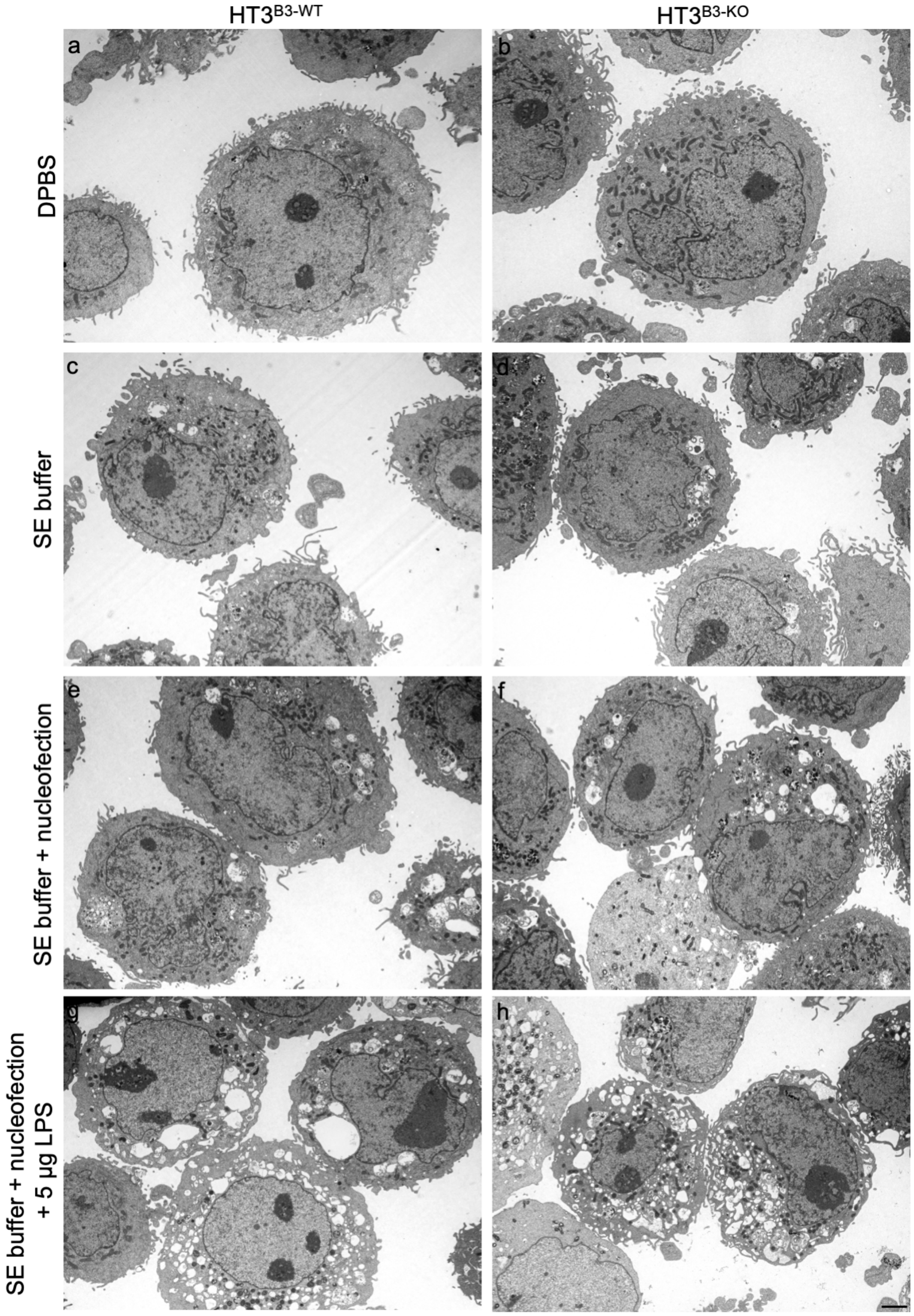
HT3B3-WT
HT3B3-KO
DPBS
SE buffer
SE buffer + nucleofection
SE buffer + nucleofection + 5 µg LPS
a
b
c
d
e
f
g
h

Supplementary Figure 19. LPS^{cyto} induces cytoplasmic vacuolization in both HT3$^{B3\text{-}WT}$ and HT3$^{B3\text{-}KO}$ cells. (a-h) Representative low power (2000x) TEM images of either HT3$^{B3\text{-}WT}$ (a, c, e, g) or HT3$^{B3\text{-}KO}$ (b, d, f, h) cells treated with DPBS (a, b), SE nucleofection buffer only (c, d), SE buffer plus nucleofection without LPS (e, f) and SE buffer plus nucleofection with 5 µg of LPS (g, h). All cells were allowed to recover in media for 2 hours. Scale bar represents 2 µm. Note the presence of cytoplasmic vacuoles in both HT3 cell lines.

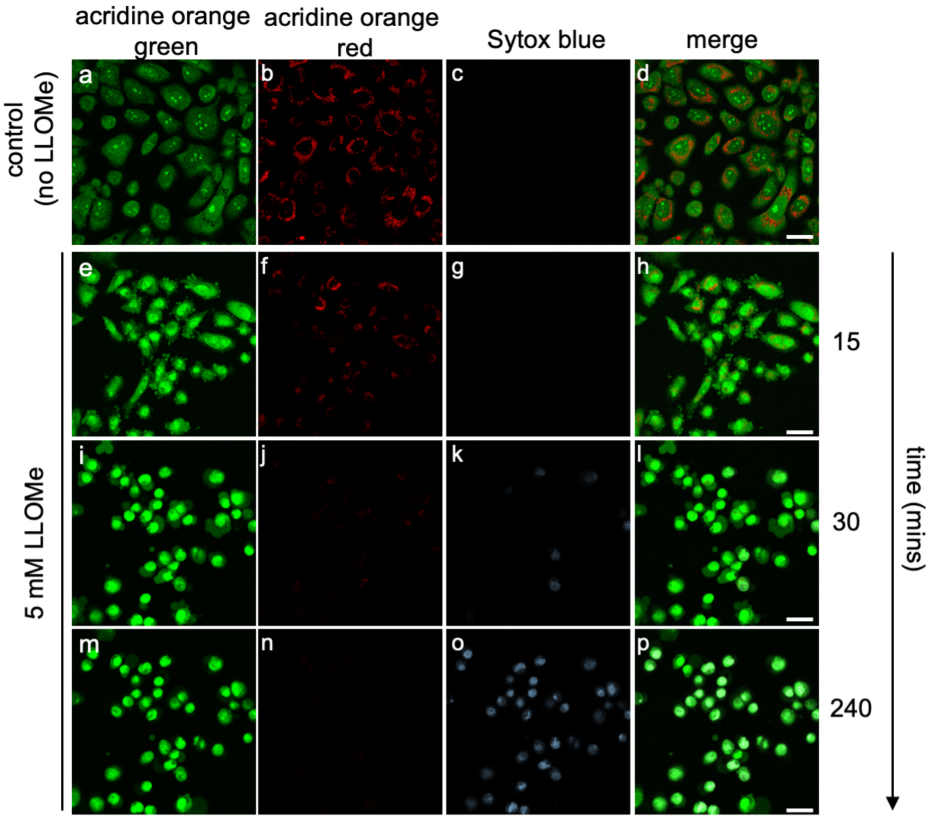
acridine orange green
acridine orange red
Sytox blue
merge
control (no LLOMe)
5 mM LLOMe
a
b
c
d
e
f
g
h
i
j
k
l
m
n
o
p
15
30
240
time (mins)

Supplementary Figure 20. LLOMe induces lysosomal membrane permeabilization in HT3 cells. (a-p) Representative confocal images of HT3$^{B3\text{-}WT}$ cells labeled with acridine orange and Sytox blue and imaged using excitation lasers 405 nm (blue; c, g, k, o), 488 nm (green; a, e, i, m) and 561 nm (red; b, f, j, n) in x, y, z over time. A merge of all channels is also shown (d, h, l, p). Cells were imaged prior to the addition of LLOMe (-a-d) and then at the indicated time points after the addition of 5 mM LLOMe (e-p). Scale bars represent 25 μm. Note the loss of red acidic vesicles with LLOMe treatment.

a

Fig. 5a

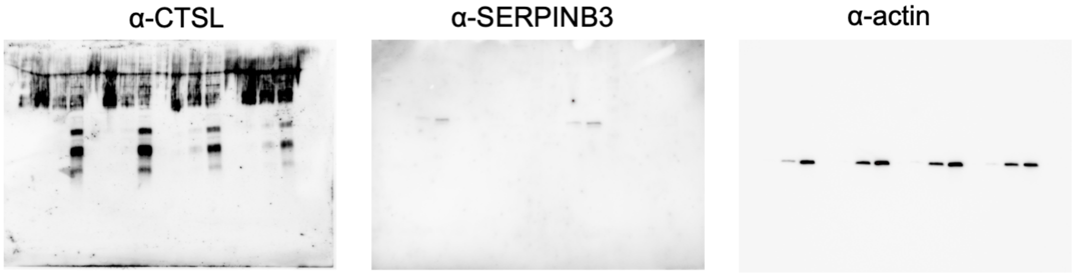

b

Fig. 6b

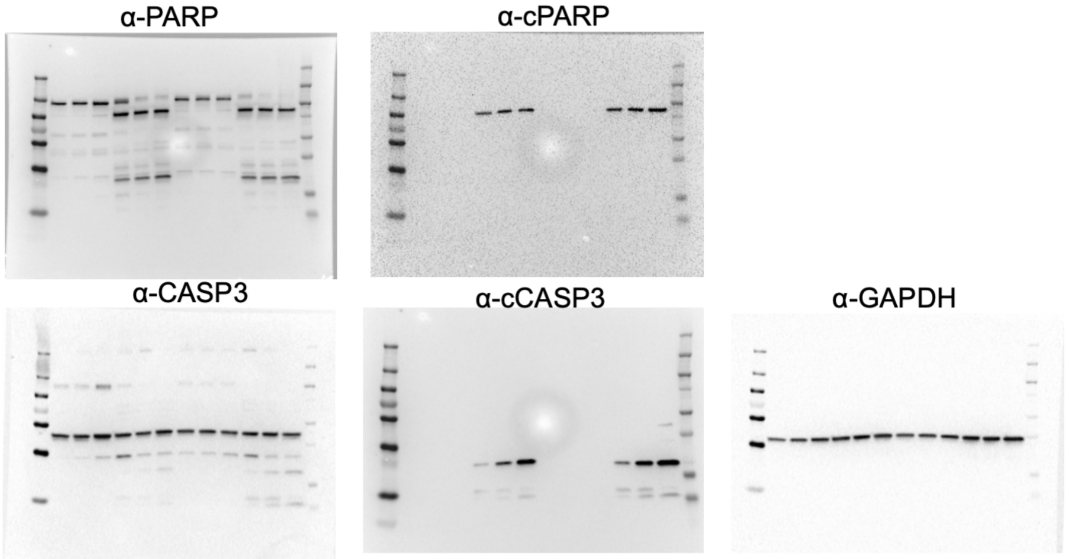

c

Fig. 9c

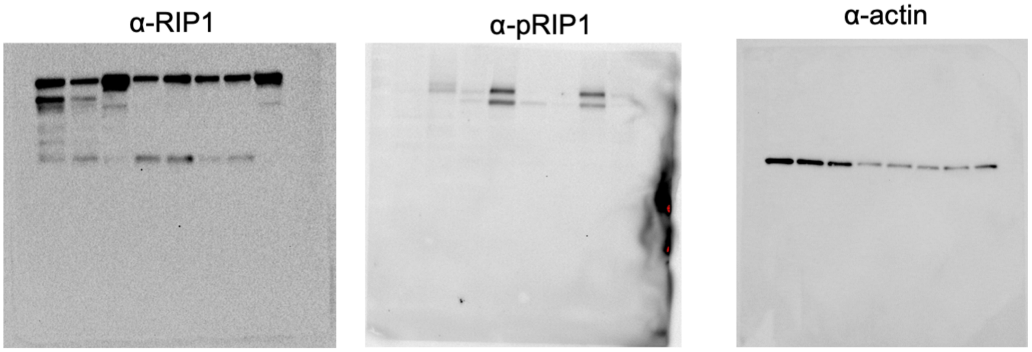

d

Fig. 10b

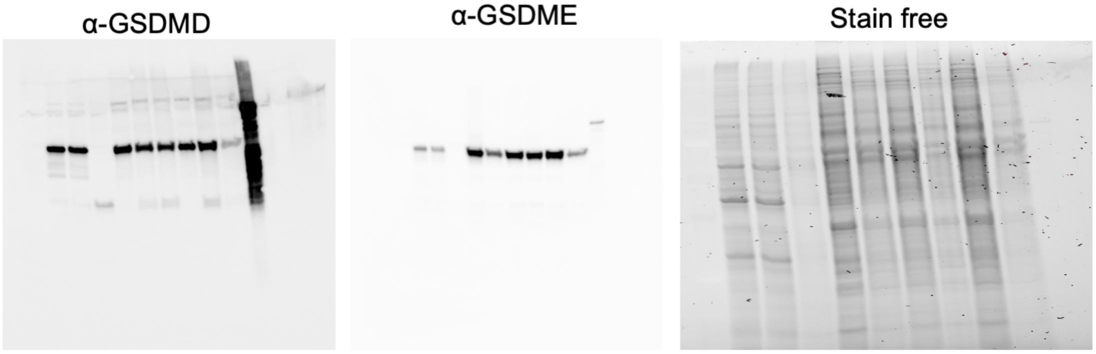

e

α-GFP α-tubulin

Fig. S1d

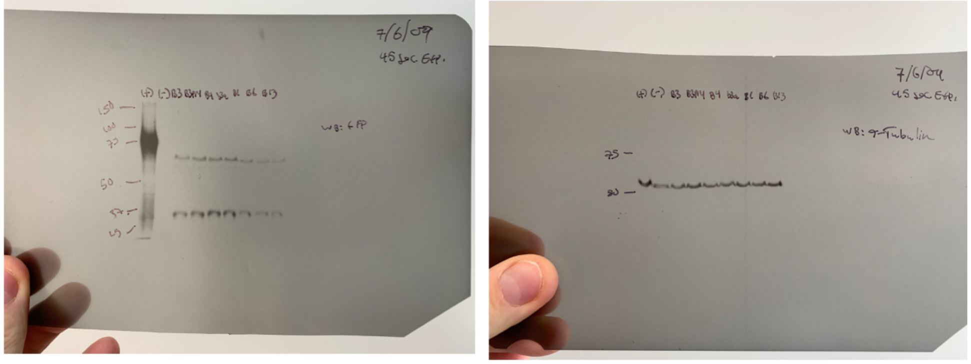

f

Fig. S3c

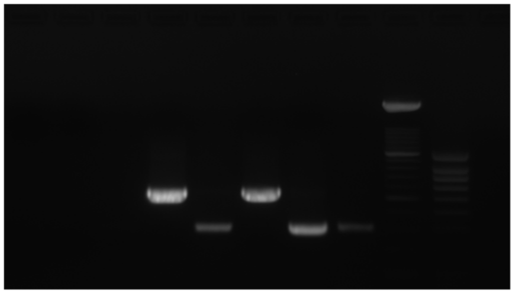

g

α-SERPINB3/4 α-SERPINB4 α-actin

Fig. S5d

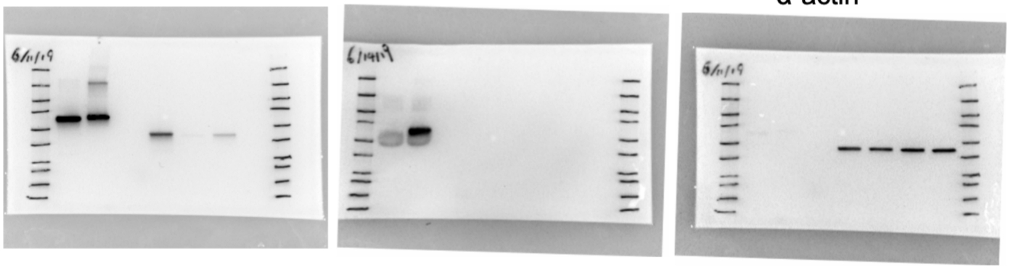

h

α-SERPINB3/4 α-SERPINB4 α-GAPDH

Fig. S7a

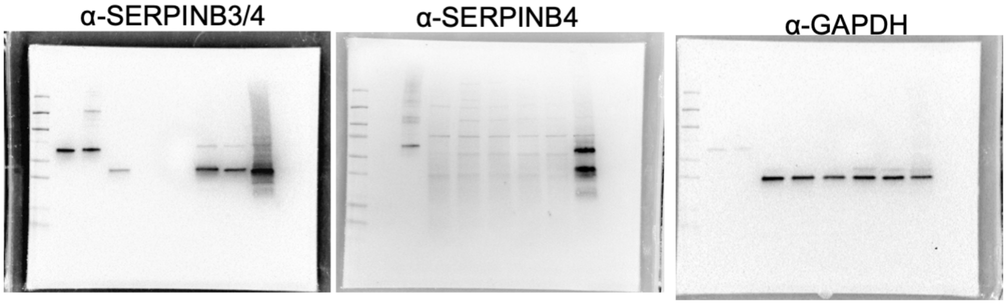

i

Fig. S8

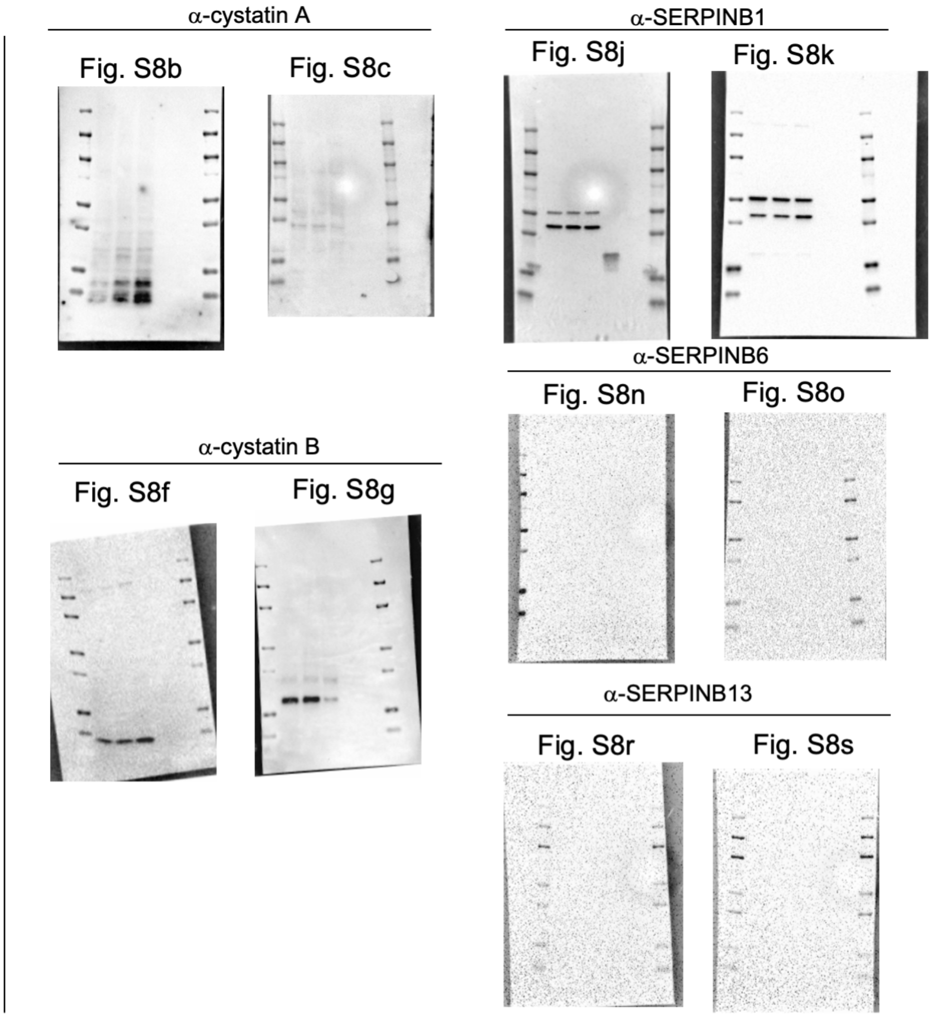

α-cystatin A
Fig. S8b
Fig. S8c
α-SERPINB1
Fig. S8j
Fig. S8k
α-SERPINB6
Fig. S8n
Fig. S8o
α-cystatin B
Fig. S8f
Fig. S8g
α-SERPINB13
Fig. S8r
Fig. S8s

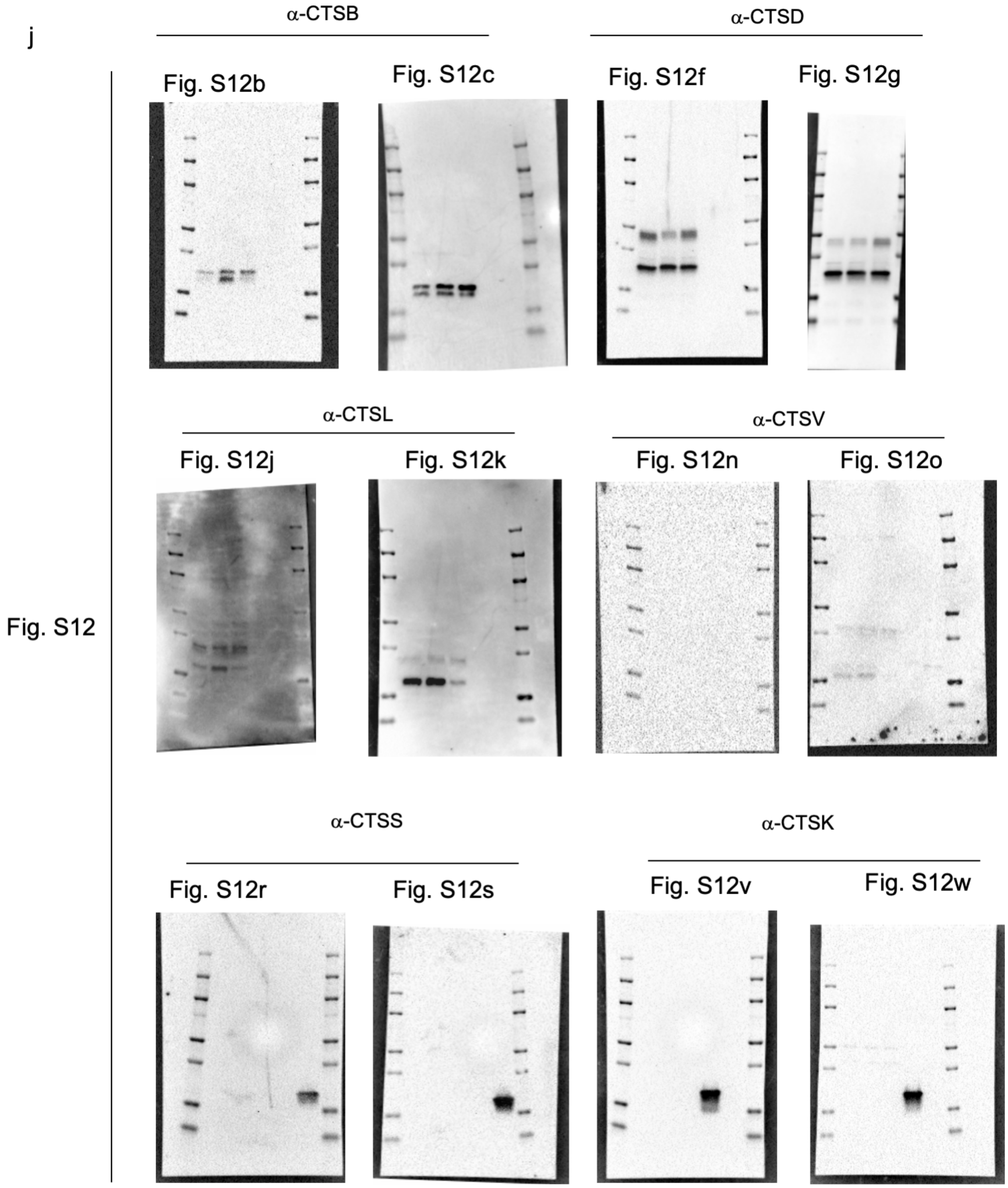
j
Fig. S12
α-CTSB
α-CTSD
Fig. S12b
Fig. S12c
Fig. S12f
Fig. S12g
α-CTSL
α-CTSV
Fig. S12j
Fig. S12k
Fig. S12n
Fig. S12o
α-CTSS
α-CTSK
Fig. S12r
Fig. S12s
Fig. S12v
Fig. S12w

α-actin

k

Figs. S8 and S12 actin blots

Figs. S8d, S12d & S12h

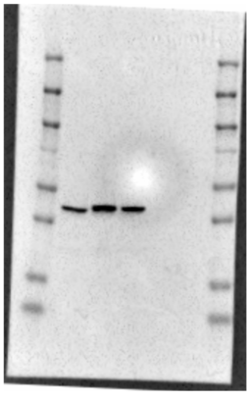

Fig. S8e

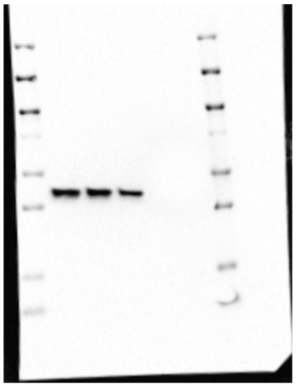

Figs. S8h, S12l & S12p

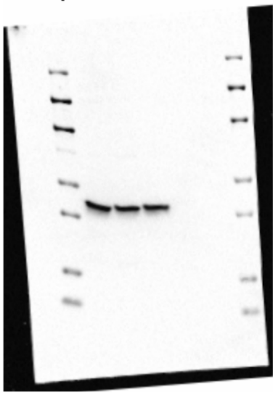

Figs. S8i, S12e, S12m & S12q

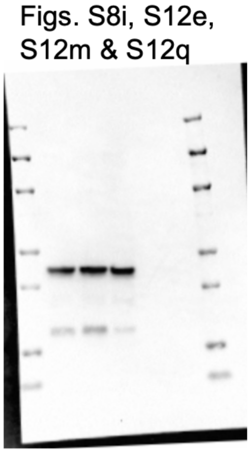

Figs. S8l & S12x

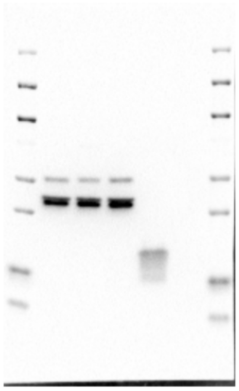

Figs. S8m & S12y

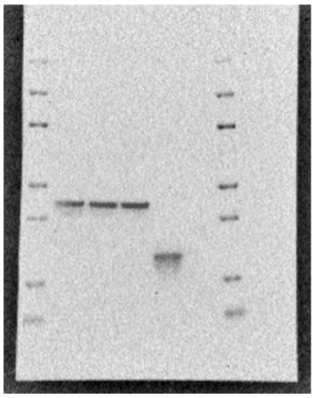

Fig. S8p

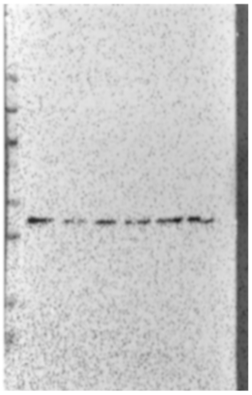

Figs. S8q & S12u

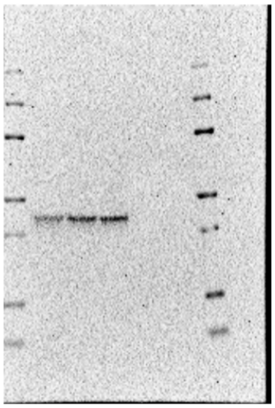

Fig. S12i

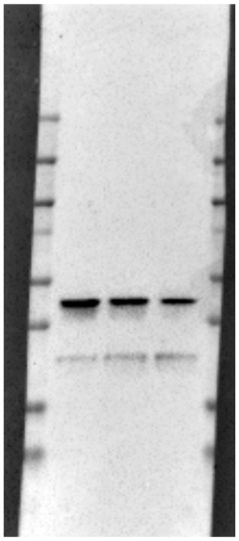

Fig. S12t

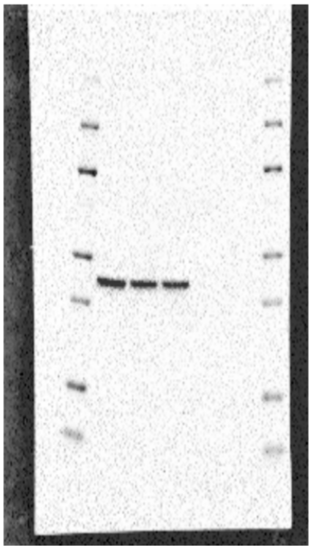

l

Fig. S13a

Stain free

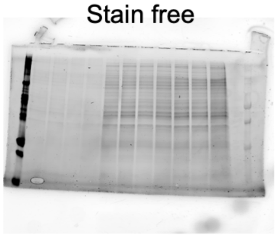

Fig. S13b

α-CTSD

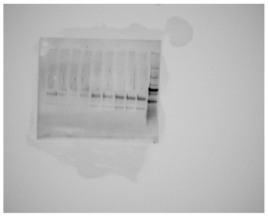

Stain free

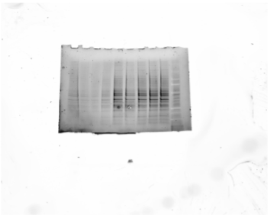

α-GAPDH

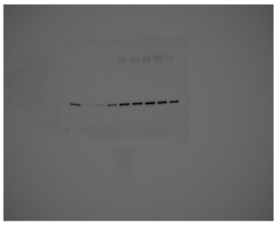

m

Fig. S17h

α-CASP1

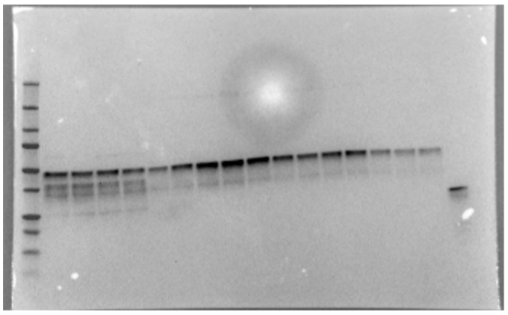

α-actin

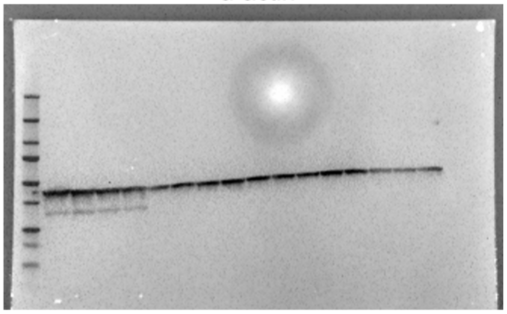

Fig. S17i

α-GSDMD

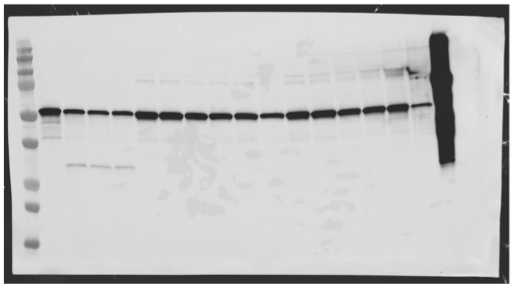

α-actin

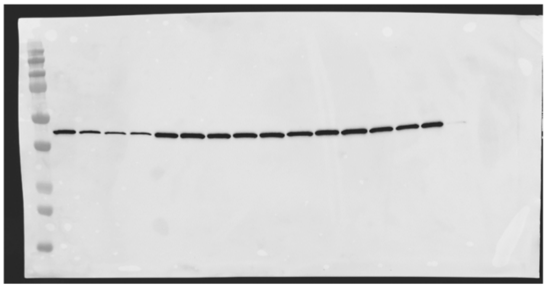

n

Fig. S18b

α-GSDMD

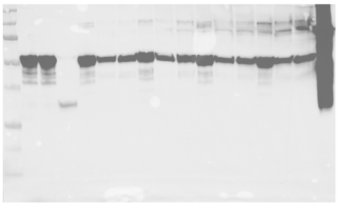

α-GSDME

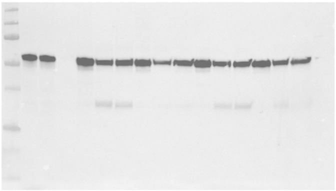

Stain free

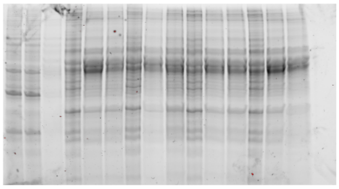

Supplementary Figure 21. Uncropped immunoblots and gel images from data used in this manuscript. (a-n) Uncropped images from Figures 5 (a), 6 (b), 9 (c), 10 (d), S1 (e), S3 (f), S5 (g), S7 (h), S8 (i, k), S12 (j, k), S13 (l), S17 (m) and S18 (n). The antibodies or stains used are indicated above the individual images. The corresponding figure panels are denoted on the left.
