# Supplementary material for: Lysoptosis is an evolutionarily conserved cell death pathway moderated by intracellular serpins
Source: Commun Biol. 2022 Jan 12;5:47. doi: 10.1038/s42003-021-02953-x (PMC8755814; doi:10.1038/s42003-021-02953-x)
Supplement: Supplementary file 3 — Description of Additional Supplementary Files [file 42003_2021_2953_MOESM3_ESM.pdf]

Description of Additional Supplementary Files

File name: Supplementary Movie 1
Description: Live-cell, confocal microscopy assessed lysosomal content (red, 10K TMR-labeled dextran) and plasma membrane permeability (green) in mSerpinb3a$^{+/+}$ FIECs treated with 100% DPBS.

File name: Supplementary Movie 2
Description: Live-cell, confocal microscopy assessed lysosomal content (red, 10K TMR-labeled dextran) and plasma membrane permeability (green) in mSerpinb3a$^{-/-}$ FIECs treated with 100% DPBS.

File name: Supplementary Movie 3
Description: Live-cell, confocal microscopy assessed lysosomal content (red, 10K TMR-labeled dextran) and plasma membrane permeability (green) in mSerpinb3a$^{+/+}$ FIECs treated with 25% DPBS.

File name: Supplementary Movie 4
Description: Live-cell, confocal microscopy assessed lysosomal content (red, 10K TMR-labeled dextran) and plasma membrane permeability (green) in mSerpinb3a$^{-/-}$ FIECs treated with 25% DPBS
