# Supplementary material for: Lysoptosis is an evolutionarily conserved cell death pathway moderated by intracellular serpins
Source: Commun Biol. 2022 Jan 12;5:47. doi: 10.1038/s42003-021-02953-x (PMC8755814; doi:10.1038/s42003-021-02953-x)
Supplement: Supplementary file 8 — Reporting Summary [file 42003_2021_2953_MOESM8_ESM.pdf]

nature portfolio

Corresponding author(s): Cliff Luke and Gary Silverman

Last updated by author(s): 11/30/2021

Reporting Summary

Nature Portfolio wishes to improve the reproducibility of the work that we publish. This form provides structure for consistency and transparency in reporting. For further information on Nature Portfolio policies, see our Editorial Policies and the Editorial Policy Checklist.

Statistics

For all statistical analyses, confirm that the following items are present in the figure legend, table legend, main text, or Methods section.

n/a	Confirmed	
☐	☒	The exact sample size (*n*) for each experimental group/condition, given as a discrete number and unit of measurement
☐	☒	A statement on whether measurements were taken from distinct samples or whether the same sample was measured repeatedly
☐	☒	The statistical test(s) used AND whether they are one- or two-sided<br>*Only common tests should be described solely by name; describe more complex techniques in the Methods section.*
☒	☐	A description of all covariates tested
☒	☐	A description of any assumptions or corrections, such as tests of normality and adjustment for multiple comparisons
☐	☒	A full description of the statistical parameters including central tendency (e.g. means) or other basic estimates (e.g. regression coefficient) AND variation (e.g. standard deviation) or associated estimates of uncertainty (e.g. confidence intervals)
☒	☐	For null hypothesis testing, the test statistic (e.g. *F*, *t*, *r*) with confidence intervals, effect sizes, degrees of freedom and *P* value noted<br>*Give P values as exact values whenever suitable.*
☒	☐	For Bayesian analysis, information on the choice of priors and Markov chain Monte Carlo settings
☒	☐	For hierarchical and complex designs, identification of the appropriate level for tests and full reporting of outcomes
☒	☐	Estimates of effect sizes (e.g. Cohen's *d*, Pearson's *r*), indicating how they were calculated

Our web collection on statistics for biologists contains articles on many of the points above.

Software and code

Policy information about availability of computer code

Data collection	N/A
Data analysis	Microscopy images were analyzed using Volocity versions 6.1.1-6.5.1 (Quorum Technologies, Inc) or LASX analysis version 3.5.5 (Leica Microsytems). Immunoblots and gel images were analyzed using Image Lab version 6.1 (Bio-Rad Laboratories, Inc.) Data and Statistical analyses were performed using GraphPad Prism versions 8.0-9.3.0 (GraphPad Software, LLC) or Excel (Microsoft). Sequencing data were analysed using MacVector version

For manuscripts utilizing custom algorithms or software that are central to the research but not yet described in published literature, software must be made available to editors and reviewers. We strongly encourage code deposition in a community repository (e.g. GitHub). See the Nature Portfolio guidelines for submitting code & software for further information.

Data

Policy information about availability of data

All manuscripts must include a data availability statement. This statement should provide the following information, where applicable:

- Accession codes, unique identifiers, or web links for publicly available datasets
- A description of any restrictions on data availability
- For clinical datasets or third party data, please ensure that the statement adheres to our policy

Original data files can be found at https://data.mendeley.com/datasets indicated at the end of each appropriate figure legend. Individual chart data can be found at https://data.mendeley.com/datasets/h4n6sz9334/draft?a=02d3eae0-0f52-4fd7-9ee2-b231f5070669. Uncropped blots and gel images can be found as Supplementary figure 21.

March 2021

Field-specific reporting

Please select the one below that is the best fit for your research. If you are not sure, read the appropriate sections before making your selection.

- [x] Life sciences
- [ ] Behavioural & social sciences
- [ ] Ecological, evolutionary & environmental sciences

For a reference copy of the document with all sections, see nature.com/documents/nr-reporting-summary-flat.pdf

Life sciences study design

All studies must disclose on these points even when the disclosure is negative.

Sample size	For live cell time course imaging studies, the maximal number of fields were calculated as the number of fields that could be successfully imaged within the time points based on hardware limitations. For live-cell single time point imaging studies by confocal microscopy, the sample size was n≥10 unless otherwise stated. For 96 well plate imaging experiments n≥3 wells were used to measure intra-assay variability unless stated. For C. elegans death assays n ≥ 20 animals per experiment.
Data exclusions	Cell imaging points were excluded if the autofocus failed or analysis of assay parameters was impossible due to loss of cells or staining. For automated imaging assays, any wells/fields of view that were out-of-focus or otherwise immeasurable were excluded from data analysis. variabilities.
Replication	All experiments were repeated multiple times (n ≥ 3) unless specifically stated.
Randomization	Assays were not randomized but separated by genotype and controlled for each experiment by having wither diluent or normal controls. Where possible, positive controls were used to ensure assay parameters were correct.
Blinding	Hypotonic stress TEM's were scored by different laboratory personnel blinded to the treatment and genotype.

Reporting for specific materials, systems and methods

We require information from authors about some types of materials, experimental systems and methods used in many studies. Here, indicate whether each material, system or method listed is relevant to your study. If you are not sure if a list item applies to your research, read the appropriate section before selecting a response.

Materials & experimental systems

n/a	Involved in the study	
[ ]	[x]	Antibodies
[ ]	[x]	Eukaryotic cell lines
[x]	[ ]	Palaeontology and archaeology
[ ]	[x]	Animals and other organisms
[x]	[ ]	Human research participants
[x]	[ ]	Clinical data
[x]	[ ]	Dual use research of concern

Methods

n/a	Involved in the study	
[x]	[ ]	ChIP-seq
[ ]	[x]	Flow cytometry
[x]	[ ]	MRI-based neuroimaging

Antibodies

Antibodies used	PolyADP-ribose polymerase (PARP) (9542), cleaved PARP (5625), caspase-3 (14220), cleaved caspase-3 (9664), caspase-8 (4790), cleaved caspase-8 (9748), β-actin (3700), RIP1 (3493), phospho-RIP1 (65746), and anti-rabbit IgG, HRP-linked secondary antibody (7074) were purchased from Cell Signaling (Danvers, MA). Gasdermin D domain-containing protein 1 (GSDMDC1) (NBP2-33422) polyclonal, cathepsin L polyclonal (AF952) and SERPINB3/4 monoclonal antibodies (NBP2-45788) were purchased from Novus Biologicals (Centennial, CO). Gasdermin E (GSDME) (ab215191) was purchased from Abcam (Cambridge, MA). Monoclonal anti-cathepsin B Antibody (H-5; sc-365558) and mouse-IgGκ BP-HRP linked secondary antibody (sc-516102) was purchased from Santa Cruz Biotechnology (Santa Cruz, CA). SERPINB3 (8H11), SERPINB4 (10C12) monoclonal antibodies and SERPINB3/B4 polyclonal were used as previously described (Cataltepe, S. et al. 2000). All primary antibodies were used at 1:1000, except the SERPINB3/B4 polyclonal and secondary antibodies were used at 1:2000 dilution. A full description of all antibodies including supplier and catalogue number is included in the methods section.
Validation	Antibodies were selected based on prior publication records (see references), manufacturer validations (see methods) and by providing the appropriate controls.

Eukaryotic cell lines

Policy information about cell lines

Cell line source(s)	ATCC or generated in house at the Washington University School of Medciine in St. Louis Genome Engineering and IPSC core

	facility
Authentication	STR profiling @ ATCC
Mycoplasma contamination	All cell lines were tested periodically for mycoplasma contamination; if positive, cells were discarded, data was not used and new cell lines obtained
Commonly misidentified lines (See ICLAC register)	None

Animals and other organisms

Policy information about studies involving animals; ARRIVE guidelines recommended for reporting animal research

Laboratory animals	C. elegans and Mouse
Wild animals	N/A
Field-collected samples	N/A
Ethics oversight	University of Pittsburgh and Washington University Animal Care and Use Committees protocols 12020207 and 19-102, respectively

Note that full information on the approval of the study protocol must also be provided in the manuscript.

Flow Cytometry

Plots

Confirm that:

- [x] The axis labels state the marker and fluorochrome used (e.g. CD4-FITC).
- [x] The axis scales are clearly visible. Include numbers along axes only for bottom left plot of group (a 'group' is an analysis of identical markers).
- [x] All plots are contour plots with outliers or pseudocolor plots.
- [x] A numerical value for number of cells or percentage (with statistics) is provided.

Methodology

Sample preparation	HT3 B3-WT and B3-KO Cells were trypsinized from multiwell plates
Instrument	MacsQuant10
Software	Data was acquired by MacsQuant software and analyzed by FlowJo software. Percentages were calculated to the nearest whole number.
Cell population abundance	Cell sorting was not used, the percent cell populations are shown in figure 10
Gating strategy	Single cell gating was used on FSC and SSC to eliminate cellular debris and multiple cell clusters

- [ ] Tick this box to confirm that a figure exemplifying the gating strategy is provided in the Supplementary Information.
